# Supplementary material for: Forecasting the prevalence of overweight and obesity in India to 2040
Source: PLoS One. 2020 Feb 24;15(2):e0229438. doi: 10.1371/journal.pone.0229438 (PMC7039458; doi:10.1371/journal.pone.0229438)
Supplement: S1 File — This file contains a detailed description of the estimation of model parameters and the procedure followed in the forecasts. (DOCX) [file pone.0229438.s001.docx]

### Estimation of incidence using the Intracohort Interpolation

A key parameter in the forecasts of future overweight and obesity prevalence in India was the incidence of overweight and obesity. I estimated incidence among individuals aged 20-49 using two cross-sections of the NFHS (2005-06 and 2015-16) as they are designed to be nationally-representative. Both of these NFHS rounds collected data on an adult’s height and weight, and reported BMI, to which I applied the WHO recommended cut-offs^1^ to assign individuals one of the following three mutually exclusive nutritional status groups: underweight/normal weight; overweight; and obese.

I used the prevalence of overweight and obesity in the two surveys, in conjunction with mortality rates calculated from the Sample Registration System (SRS) to estimate the net rate of incidence of overweight and obesity using the iterative intra-cohort interpolation procedure^2^ developed by Stupp (1988). Using this procedure, I converted the changes in the prevalence of overweight and obesity among specific cohorts into to age-specific transition rates using an iterative procedure. Assuming that age-specific incidence is constant over the inter-survey period (in our case, 10 years), the final iteration provides age-specific incidence that has the highest likelihood of resembling the actual changes to each cohort^2^.

In order to calculate the rates in the inter-survey period, for men and women in urban and rural areas separately, I required the following three inputs:

- The prevalence of overweight and obesity in 2005-06 and 2015-16 separately for men and women in urban and rural areas by five-year age groups.
- The central age-specific mortality rates in 2005 and 2015 separately for men and women in urban and rural areas.
- The relative risk of dying for individuals classified as overweight relative to the underweight/normal weight category; and the relative risk of dying for individuals who are obese relative to overweight.

In brief, the procedure is carried out using the following steps:

Calculate the cohort incidence rates *I_k_(c)*, indicating the change in status (in our case nutritional status) occurring to cohort *c*, during the inter-survey period.

$I_{k}\left( c \right)=F_{k}\left( c,c+T \right)-F_{k}(c,c)$ for $c=\alpha to \beta-T$

Equation 1

Where *F_k_(c,c)* and *F_k_(c,c+T)* refer to the prevalence of the nutritional status, *k*, in question amongst age cohort *c* in the initial period and the succeeding survey, respectively, with *T* denoting the length of the inter-survey period. The cohorts $\alpha\mathrm{and}\beta$ refer to the youngest and oldest cohort for which initial data is available. If *P_k_* is the proportion that have not yet experienced nutritional status *k*, one can rewrite the equation above as:

$$I_{k}(c)={ln[P}_{k}(c,c)]-{ln[P}_{k}(c,c+T)]$$

Equation 2

One can then obtain an initial estimate of the age-schedule by weighting the cohort rates (*I_k_(c)*) by the amount of time spent in a particular age-group. For instance, when calculating the rates for the 25-29 ages group, I added the product of the cohort incidence rate for the 15-19-year age group and the time spent they spent aged 25-29 years in the 10 years following the initial survey (2.5 years), to the product of the cohort rate among 20-24 year olds and the time spent aged 25-29 this cohort (5 years), and the product of the cohort rate among 25-29 year olds and the time spent aged 25-29 this cohort (2.5 years). This summation is subsequently divided by the length of the inter-survey period (10 years). Finally, I adjusted this calculation by adding the difference in the mortality rates between those with and without nutritional status *k*, in order to account for the fact that they are likely to die at different rates. Formally:

$f_{0}\left( a \right)=\frac{\sum_{a-T}^{a} I_{k}\left( c \right)*p\left( c,a \right)}{\sum_{c}^{c+T} p\left( c,a \right)}+[m_{k}(a)-m_{!k}(a)]$

Equation 3

Using the example of the first iteration of the rate for age group 25-29, successive iterations of the age-specific incidence rate are calculated in the following way:

1. Calculate the products of the cohort incidence rate for each cohort passing through age-group 25-29 with the initial age-specific rate estimate. Subsequently, calculate a weighted sum of these products by the time spent by each cohort in the five-year age-group for which the rate is required. For the age group 25-29 years between surveys ten years apart, this is calculated as follows:

$${[I}_{k}\left( 15-19 \right)*f_{0}\left( 20-24 \right)*2.5]+{[I}_{k}\left( 20-24 \right)*f_{0}(25-29)*5]+$$

$${[I}_{k}\left( 25-29 \right)*f_{0}\left( 30-34 \right)*2.5]$$

1. Calculate the sum of the age-specific rates from iteration 0, weighted by the time spent in each age group in the inter-survey period:

$$\left[ f_{0}\left( 20-24 \right)*2.5 \right]+[f_{0}(25-29)*5]+$$

$$[f_{0}\left( 30-34 \right)*2.5]$$

1. Divide the sum obtained in step 1 by the sum obtained in step 2, and as in Equation 3, adjust this by adding the difference in the mortality rates between those with and without nutritional status *k*.

Table 1 and Table 2 present an example of the calculation of age-specific annualised incidence of obesity among the overweight population of women in urban areas. In the table of inputs (Table 1), the first column refers to the age groups; the second and third contain the proportion *not* having experienced event *k* in 2005 and 2015, i.e. the proportion of the population who are overweight among those who are either overweight or obese. The fourth column contains the geometric average prevalence of overweight, which is used in the calculation of the average difference between the mortality rates of the obese and overweight populations. The fifth and sixth columns contain age-specific central death rates in 2005 and 2015 (expressed per 100,000-person years) estimated from SRS lifetables, the seventh column contains the age-specific relative risks of dying among obese women, relative to overweight women, and the final column contains the average difference in age-specific mortality between the obese and overweight population.

Table 1. Inputs needed to calculate obesity incidence using the Iterative Intracohort Interpolation method (women in urban areas)

| *Age group (years)* | *F (2005)* | *F (2015)* | *S*(a)* | *m(a) 2005* | *m(a) 2015* | *RR** | *Δ m(a)* |
| --- | --- | --- | --- | --- | --- | --- | --- |
| *15-19* | 0.878236 | 0.791850 | 0.833925 | 0.001188 | 0.000885 | 1.165 | 0.000166 |
| *20-24* | 0.827412 | 0.782315 | 0.804548 | 0.001393 | 0.000998 | 1.165 | 0.000191 |
| *25-29* | 0.789383 | 0.756977 | 0.773010 | 0.001463 | 0.000885 | 1.165 | 0.000187 |
| *30-34* | 0.766696 | 0.721596 | 0.743804 | 0.001439 | 0.000894 | 1.165 | 0.000185 |
| *35-39* | 0.719382 | 0.690802 | 0.704947 | 0.001747 | 0.001228 | 1.165 | 0.000234 |
| *40-44* | 0.678422 | 0.675490 | 0.676954 | 0.002184 | 0.001712 | 1.165 | 0.000305 |
| *45-49* | 0.696181 | 0.668257 | 0.682077 | 0.003491 | 0.002601 | 1.165 | 0.000478 |

Table 2, is calculated using the formulae above and shows that the age-schedule of age-specific overweight incidence converges after approximately three iterations.

Table 2. Iterations of the intracohort interpolation using inputs from Table 1 (expressed per person-year) (5 d.p)

| *Age group (years)* | *I(c)* | *f_0_(a)* | *f_1_(a)* | *f_2_(a)* | *f_3_(a)* |
| --- | --- | --- | --- | --- | --- |
| *15-19* | 0.01486 | 0.01502 | 0.01502 | 0.01502 | 0.01502 |
| *20-24* | 0.01368 | 0.01466 | 0.01467 | 0.01467 | 0.01467 |
| *25-29* | 0.01334 | 0.01408 | 0.01409 | 0.01409 | 0.01409 |
| *30-34* | 0.01267 | 0.01344 | 0.01347 | 0.01347 | 0.01347 |
| *35-39* | 0.00737 | 0.01174 | 0.01181 | 0.01181 | 0.01181 |

This procedure was repeated for men and women in urban and rural areas aged 20-49 years. Separate sets of incidence rates were calculated for the transition from underweight/normal weight to overweight, and overweight to obesity.

One limitation of this procedure is that it calculates net transition rates, rather than gross rates, i.e. calculates the overall flow from state *!k* to *k*, rather than separate gross rates in and out of the nutritional states. In order to accommodate this in our final model, fixed remission rates were introduced on the rates of transition back to lower weight classes. These were added to the net rates of transition to overweight and obesity to convert the net rates into gross ones.

### Calculation of the incidence of overweight and obesity in old age

For individuals aged 50-69 years, I calculated the number of incident cases, over all ages, of overweight and obesity among the population classified as initially underweight/normal weight and overweight, respectively, using longitudinal data from the Study on Global Ageing and Adult Health (SAGE) waves 0 (2002-04) and 1 (2007-10). This calculation was carried out for men and women separately. In order to estimate the approximate person-time at risk of transitioning, I assumed that incident cases occurred at the halfway-point between the waves.

As there were too few incident cases to reliably estimate age-specific rates directly from these data, I used indirect standardisation to estimate risk in India and obtained the detailed age pattern of incidence from an external standard. I identified a study conducted in the United States that estimated the following age-pattern of obesity incidence using data from the Behavioural Risk Factor Surveillance System^3^:

Table 3. Incidence of obesity in the United States reported in Pan et al (2011)

| *Age group (years)* | *Incidence rate (%)* |
| --- | --- |
| *18-29* | 6.4 |
| *30-49* | 4.8 |
| *50-59* | 3.3 |
| *70+* | 1.5 |

In order to obtain rates for the age-groups I needed (50-54; 55-59; 60-64; and 64-69), I fitted a spline to the above data, which I refer to as the standard rates, assuming that the above incidence rates pertained to the mid-point of each age-group.

Indirect standardisation involved scaling these standard rates by a standardised incidence ratio (SIR), which is defined as the ratio of the observed number of incident cases in India (estimated using SAGE data) to the expected number of events (how many incident cases we could expect to see if the standard rates was exposed to the same amount of person-time at risk as in the Indian data). Confidence intervals (95%) were obtained by multiplying the standard rates for the 50-54; 55-59; 60-64; and 64-69 age groups by upper and lower bounds of the SIR, which were obtained in the following way:

$${SIR}_{95\%ci}=SIR\pm(1.96*\frac{\sqrt{O}}{E})$$

Equation 4

Where *O* and *E* refer to the number of observed and expected incident cases, respectively. After obtaining a set of incidence rates of overweight and obesity among men and women, I obtained urban- and rural-specific incidence estimates using a scalar of the average association between urban and rural incidence and overall incidence that I estimated using the NFHS data.

### Forecasting the central mortality rate using the Lee-Carter model

Rather than remaining constant over the forecast period, the age-specific mortality rates of the Indian population are likely to decline. Failure to account for future declines in mortality is likely to produce underestimates of the future prevalence of overweight and obesity.

In order to forecast future age–specific mortality, I used the Lee-Carter model^4–6^. This model summarises a series of historic age-specific mortality schedules over time using the following three parameters^7^:

Two age-specific (*a*) parameters:

- The average historic log age-specific mortality schedule (*a_a_*)
- Age-specific deviations from this centred log mortality schedule (*b_a_*)

One time-specific (*t*) parameter:

- Overall level of mortality ($\kappa$*_t_*).

The Lee-Carter model proposes that any set of age-specific log mortality rates can be summarised as follows:

$$\log\left( m_{a,t} \right)=a_{a}+b_{a}\kappa_{t}$$

Equation 5

Fitting the model involves subtracting the average log mortality curve (*a_a_*) from the log mortality rates and performing a singular value decomposition (SVD) on the resulting centred mortality surface. From the SVD one can calculate *b_a_* and *K_t_* as follows^7^:

$b_{a}=\frac{v}{\sum v}$ and $\kappa_{t}=sU\sum v$

Equation 6

Where *v* and *u* refer to the left and right singular vectors that correspond to the largest singular value (*s*).

The Lee-Carter model forecast depends entirely on the extrapolation of the $\kappa$*_t_* parameter which I obtained using a random walk with a drift, which is estimated using maximum likelihood in ARIMA (0,1,0):

$$\tilde{d}=\frac{\kappa_{T}-\kappa_{1}}{T-1}$$

Equation 7

The drift parameter is used to predict the mortality schedule in all succeeding periods after the last year for which we have recorded mortality data.

I obtained input mortality data for the Lee-Carter mortality forecast from the SRS, which contains lifetable data for every year from 1997 to 2013 for men and women separately in both urban and rural India. Using the *_n_q_a_* values contained in the abridged lifetables, which denotes the conditional probability that an individual aged *a* will die between exact ages *a* and *a+n*, I calculated age-specific mortality rates using a rearrangement of Chiang’s (1968) formula^8,9^ This formula was initially designed to calculate this conditional probability using age-specific mortality rates. Specifically,

$${{}_{n}^{k}m}_{a}=\frac{{{}_{n}^{k}q}_{a}}{n+\left( {}_{n}^{k}{{a'}_{a}}-n \right){}_{n}^{k}{q_{a}}}$$

Equation 8

Where *k* refers to the subpopulation our central mortality rate relates to, i.e. urban men, urban women, rural men, or rural women. Additionally, *_n_a’_a_* refers to the proportion of the interval lived by those who die between ages *a* and *a+n*. The parameter values obtained from the series of age-specific mortality schedules from 1997 to 2013 are shown in Figure 1 using the example of women in urban areas.

Using these parameters, I forecasted future mortality using the **forecast** function in R, which also produced 95% confidence intervals^10,11^. Figure 2 shows the forecasted log mortality rates for the ages of interest between 1997 and 2050.

Figure 1. Plot of the a_a_ (Panel 1), b_a_ (Panel 2) and k_t_ (Panel 3) parameters used in the Lee-Carter Mortality forecast (Women in Urban India aged 20-69 years)


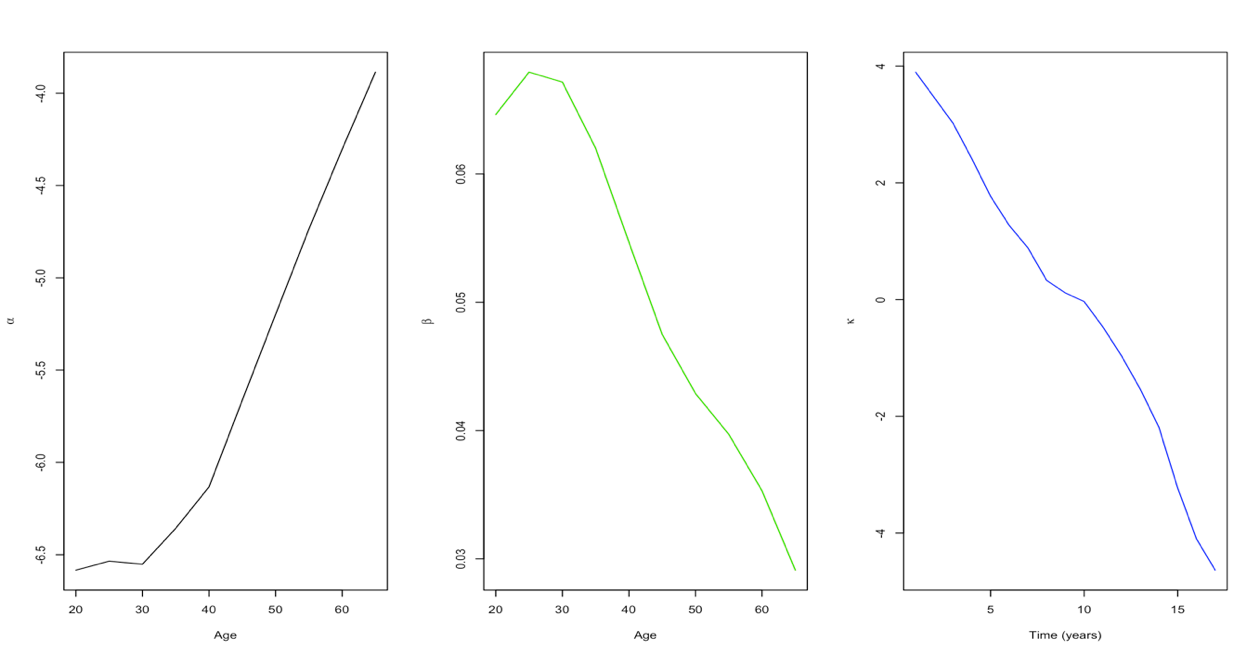


Figure 2. Age-specific log mortality rates (20-69 years) among women in urban India (1997-2013 actual; 2014-2050 forecast)


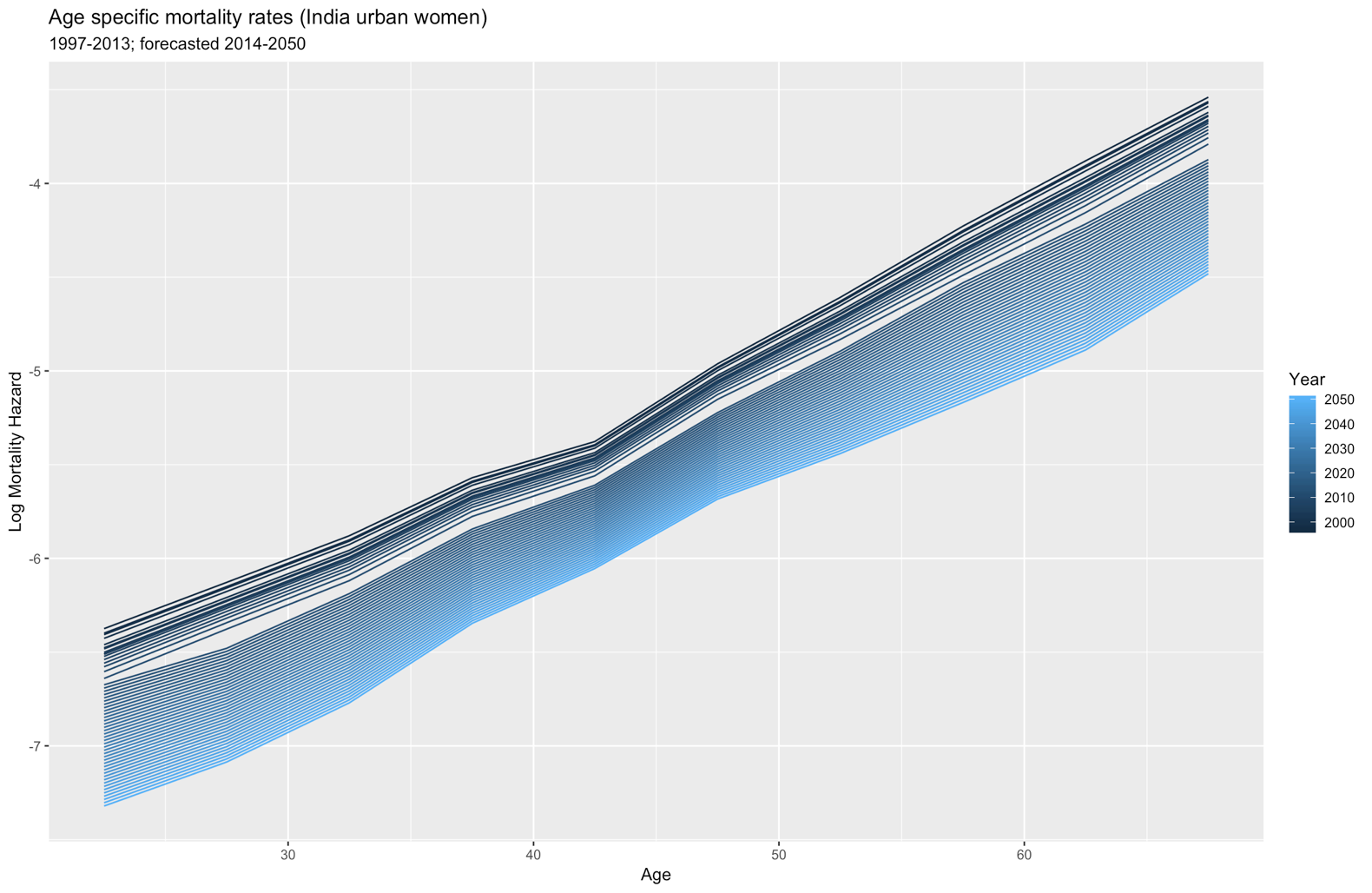


*Source: SRS Abridged lifetables

### Differential mortality by nutritional status

Individuals classified as underweight/normal weight, overweight, and obese are all likely to have different risks of mortality, and failure to account for this may bias my final estimates of the future prevalence of various nutritional statuses. Using relative risks of mortality for different BMI categories, relative to a specified baseline, one can adjust the central mortality rate to obtain BMI group-specific mortality rates. I used the relative risks of dying by BMI group, relative to the normal weight category, reported in Pednekar et al. (2008)^12^. This study examined the association of body weight with mortality in Mumbai in a prospective cohort study that followed up 148,173 individuals aged 35 and above, recruited in 1991-97 through to 1997-2003. As the study reported relative risks for men and women aged 35-59 and 60+, we used the relative risks for the 35-59 age group for individuals aged 20-34 years.

Table 4. Relative risks of dying by BMI group in India (reference group: Normal weight 18.5-24.9kg/m^2^)

| *BMI Group* | *Age (years)* | *Women* | | | *Men* | | |
| --- | --- | --- | --- | --- | --- | --- | --- |
|  |  | *RR* | *Lower* | *Upper* | *RR* | *Lower* | *Upper* |
| *Underweight* | *35-59* | 1.67 | 1.45 | 1.93 | 1.46 | 1.87 | 2.18 |
|  | *60+* | 2.02 | 1.33 | 1.6 | 1.35 | 1.28 | 1.44 |
| *Overweight* | *35-59* | 1.03 | 0.88 | 1.2 | 0.89 | 0.8 | 0.98 |
|  | *60+* | 0.82 | 0.72 | 0.93 | 0.87 | 0.8 | 0.94 |
| *Obese* | *35-59* | 1.2 | 0.95 | 1.51 | 1.22 | 1.01 | 1.48 |
|  | *60+* | 0.83 | 0.66 | 1.03 | 0.93 | 0.77 | 1.13 |

Source: Pednekar et al (2008) page 528

I obtained separate mortality rates for individuals in the obese and overweight BMI categories and updated them at every interval as the apportioning of the central mortality rate to various BMI categories is dependent on the proportion of the population in those categories. For example, I obtained the mortality rate of the obese population in the following way:

1. Obtain the relative risk of dying for the obese population relative to the non-obese population.

$${{}_{!OB}^{OB}R}_{a,t}=\frac{{{}_{N}^{OB}R}_{a,t}}{{({}_{N}^{UW}R}_{a,t}*{}_{-OB}^{UW}{\theta_{a,t}})+{({}_{N}^{OW}R}_{a,t}*{}_{-OB}^{OW}{\theta_{a,t}})+{}_{-OB}^{N}{\theta_{a,t}}}$$

Equation 9

Where ${{}_{N}^{OB}R}_{a,t}$, ${{}_{N}^{UW}R}_{a,t}$, and ${{}_{N}^{OW}R}_{a,t}$ represent the relative risks of dying among the population who are obese, underweight, and overweight, respectively, relative to the normal weight reference category, reported in Table 4. The terms ${}_{-OB}^{OW}{\theta_{a,t}}$, ${}_{-OB}^{UW}{\theta_{a,t}}$, and ${}_{-OB}^{N}{\theta_{a,t}}$ denote the proportion of the total population that excludes the obese population, who are overweight, underweight and normal weight, respectively.

1. Obtain the mortality rate among the non-obese population.

$$m_{a,t}^{!OB}=\frac{\bar{m}_{a,t}}{(\gamma_{a,t}^{OB}*{{}_{!OB}^{OB}R}_{a,t})-\gamma_{a,t}^{OB}+1}$$

Equation 10

Where $\bar{m}_{a,t}$ and $\gamma_{a,t}^{OB}$denote the central death rate and the prevalence of obesity, respectively, for age group *a* at time *t*.

1. Use the mortality rate among the non-obese population to obtain the mortality rate among the obese population, using the following formula:

$$m_{a,t}^{OB}=m_{a,t}^{!OB}*{{}_{!OB}^{OB}R}_{a,t}$$

Equation 11

These calculations were repeated to obtain the relative risk of dying among the overweight population relative to those who are not.

The mortality rate among the underweight/normal weight population was subsequently obtained with the knowledge that the central death rate ($\bar{m}_{a,t})$ is a weighted sum of the death rates in each of the *k* lifetables. Specifically,

$$m_{a,t}^{UW/N}=\bar{m}_{a,t}-\left( m_{a,t}^{OB}*\gamma_{x,t}^{OB} \right)+ (m_{a,t}^{OW}*\gamma_{a,t}^{OW})$$

Equation 12

### Calculation of multi-status lifetables

The incidence-based model used to forecast the future prevalence of overweight and obesity in India is presented in the main paper. In brief, the future predicted prevalence of overweight and obesity in urban and rural India are determined by a set of age-specific rates of flow into these BMI groups, which I refer to as health states, rates of flow out of these groups back to lower BMI groups, rates of urbanisation and age- and health state- specific mortality rates.

A common way of forecasting in this framework is to populate a matrix of transition rates before converting it to a matrix of transition probabilities. In this case, each cell of a transition matrix denotes the probability that an individual will be in a particular health state in a succeeding period, dependent on the state in which they started. However, most studies apply transition probabilities to the population at risk of a transition *at the beginning* of a time period to determine the distribution of the population across health states in a succeeding time period. Importantly, the models do not take account of a changing population-at-risk within a time period, for instance, allowing people to follow various pathways within a five-year forecast step, albeit determined by discrete age-period rates. This may become more problematic when working with relatively wider age-groups. In order to fully account for this, I employed a multi-state lifetable system developed by Schoen and Nelson (1974) who addressed questions about flows in and out of marriage in the UK and USA using a system of lifetables^13^. Rather than work with transition probabilities derived from the rates, this model directly uses the rates to calculate the forecast.

The forecast was carried out using a system of six interconnected increment-decrement lifetables; one for each of the transient health states (for example, overweight (rural) and obese (urban)). If the number of individuals alive at age *a* in state *k* is $l_{a}^{k}$, then the number of individuals in state *k* in age group *a+n*, whereby *n* denotes the interval width, can be formally written as:

$$l_{a+n}^{k}=l_{a}^{k}+\sum_{i\neq k}^{k} {{}_{n}^{i}d}_{a}^{k}-\sum_{i\neq k}^{k} {{}_{n}^{k}d}_{a}^{i}-{}_{n}^{k}{d_{a}^{m}}$$

Equation 13

Whereby $\sum_{i\neq k}^{k} {{}_{n}^{i}d}_{a}^{k}$ refers to the total number of decrements from state *i* to state *k*, $\sum_{i\neq k}^{k} {{}_{n}^{k}d}_{a}^{i}$ refers to the total number of decrements from state *k* to other states *i*, and ${}_{n}^{k}{d_{a}^{m}}$ the total number of deaths from state *k* between exact ages *a* and *a+n*. For instance, in this system, the population of obese women in urban India in 2015 will depend on the number of women entering this state from the overweight rural, overweight urban and obese rural lifetables between 2010 and 2015, the number exiting to the overweight urban state between 2010 and 2015, and the number of deaths among urban obese women between 2010 and 2015.

The total person-years of exposure for a cohort in lifetable *k* between ages *a* and *a+n* to a particular decrement is denoted by the standard lifetable notation ${{}_{n}^{k}L}_{a}$. The number of decrements needed to calculate the total number of entries in and out of a particular health state is therefore:

$${}_{n}^{i}{d_{a}^{k}={{}_{n}^{i}r}_{a}^{k}{* {}_{n}^{k}L}_{a}}$$

Equation 14

Where *r* represents the rate of transition (incidence, mortality, remission or urbanisation).

After repeating the above equation for the total number of transitions between ages *a* and *a+n*, and substituting the values into $l_{a+n}^{k}$ to obtain an initial estimate of the number of individuals in state *k* at age *a+n*, I obtained an updated estimate of the number of person-years of exposure using the formula below, which expresses the total number of person-years in terms of initial and subsequent people in each health state.

$$\hat{{{}_{n}^{k}L}_{a}}=\frac{n}{2}(l_{a}^{k}{+l}_{a+n}^{k})$$

Equation 15

Using this new estimate of person-years of exposure to a particular decrement, I recalculated the number of decrements expected and the number of individuals alive at age *a+n* in each lifetable *k*. I repeated this iterative procedure until the $\hat{{{}_{n}^{k}L}_{a}}$converged, ensuring that the appropriate population at risk of a decrement was considered at each time step of the forecast.

1. WHO. Obesity and Overweight: Fact sheet 311 [Internet]. WHO Media Centre. 2016 [cited 2017 Jul 13]. Available from: http://www.who.int/mediacentre/factsheets/fs311/en/

2. Stupp PW. Estimating Intercensal Age Schedules by Intracohort Interpolation. Popul Index. 1988;54(2):209–24.

3. Pan L, Freedman DS, Gillespie C, Park S, Sherry B. Incidences of obesity and extreme obesity among US adults: Findings from the 2009 Behavioral Risk Factor Surveillance System. Popul Health Metr. 2011;9(1):56.

4. Lee RD, Carter LR. Modeling and forecasting U.S. mortality. J Am Stat Assoc. 1992;87(419):659–71.

5. Yadav A, Yadav S, Kesarwani R. Decelerating Mortality Rates in Older Ages and its Prospects through Lee-Carter Approach. PLoS One. 2012;7(12):e50941.

6. Lee R, Miller T. Assessing the performance of the Lee-Carter approach to modeling and forecasting mortality. In: Annual Meeting of the Population Association of America. 2000.

7. Lenart A. Lee-Carter forecasting. In: Biodemography MPOC on the, of Aging, editors. EDSD mathematical demography. Rome; 2015.

8. Preston SH, Heuveline P, Guillot M. Demography, Measuring and Modeling Population Processes. Population. 2000.

9. Chiang C. An Introduction to Stochastic Processes in Biostatistics. New York: Wiley; 1968.

10. Hyndman R, Athanasopoulos G, Bergmeir C, Caceres G, Chhay L, O’Hara-Wild M, et al. forecast: Forecasting functions for time series and linear models. R package version 8.7. 2019.

11. Hyndman RJ, Khandakar Y. Automatic Time Series Forecasting: The forecast Package for R. J Stat Softw. 2008;26(3):1–22.

12. Pednekar MS, Hakama M, Hebert JR, Gupta PC. Association of body mass index with all-cause and cause-specific mortality: Findings from a prospective cohort study in Mumbai (Bombay), India. Int J Epidemiol. 2008;37(3):524–35.

13. Schoen R, Nelson V. Marriage, divorce, and mortality: a life table analysis. Demography. 1974;11(2):267–90.
