# Supplementary material for: Forecasting the prevalence of overweight and obesity in India to 2040
Source: PLoS One. 2020 Feb 24;15(2):e0229438. doi: 10.1371/journal.pone.0229438 (PMC7039458; doi:10.1371/journal.pone.0229438)
Supplement: Attachment — Submitted filename: PONE-D-19-17017_review_cw.pdf [file pone.0229438.s003.pdf]

PLOS ONE

Forecasting the Prevalence of Overweight and Obesity in India to 2040

--Manuscript Draft--

**Manuscript Number:**	PONE-D-19-17017
**Article Type:**	Research Article
**Full Title:**	Forecasting the Prevalence of Overweight and Obesity in India to 2040
**Short Title:**	Forecasting Overweight and Obesity prevalence in India
**Corresponding Author:**	Shammi Luhar, PhD<br>London School of Hygiene and Tropical Medicine Department of Population Health<br>London, UNITED KINGDOM
**Keywords:**	Overweight; Obesity; India; Modelling study; Urban; Rural; Forecast
**Abstract:**	Background: In India, the prevalence of overweight and obesity has increased rapidly in recent decades. Given the association between overweight and obesity with many non-communicable diseases, forecasts of the future prevalence of overweight and obesity can help inform policy in a country where around one sixth of the world's population resides.<br><br>Methods<br><br>We used a system of multi-state life tables to forecast overweight and obesity prevalence among Indians aged 20-69 years by age, sex and urban/rural residence to 2040. We estimated the incidence and initial prevalence of overweight using nationally representative data from the National Family Health Surveys 3 and 4, and the Study on global AGEing and adult health, waves 0 and 1. We forecasted future mortality, using the Lee-Carter model fitted life tables reported by the Sample Registration System, and adjusted the mortality rates for Body Mass Index using relative risks from the literature.<br><br>Results<br><br>The prevalence of overweight will more than double among Indian adults aged 20-69 years between 2010 and 2040, while the prevalence of obesity will triple. Specifically, the prevalence of overweight and obesity will reach 30.4% (26.5%-34.6%) and 9.6% (5.6%-13.6%) among men, and 27.4% (23.9%-30.9%) and 13.9% (10.0%-17.5%) among women, respectively, by 2040. The largest increases in the prevalence of overweight and obesity between 2010 and 2040 is expected to be in older ages, and we found a larger relative increase in overweight and obesity in rural areas compared to urban areas. The largest relative increase in overweight and obesity prevalence was forecast to occur at older age groups.<br><br>Conclusion<br><br>The overall prevalence of overweight and obesity is expected to increase considerably in India by 2040, with substantial increases particularly among rural residents and older Indians. Detailed predictions of excess weight are crucial in estimating future non-communicable disease burdens and their economic impact.
**Order of Authors:**	Shammi Luhar, PhD
	Ian M. Timæus
	Rebecca Jones
	Solveig Cunningham
	Shivani A. Patel
	Sanjay Kinra
	Lynda Clarke
	Rein Houben

Powered by Editorial Manager® and ProduXion Manager® from Aries Systems Corporation

Additional Information:	
**Question**	**Response**
**Financial Disclosure**<br><br>Enter a financial disclosure statement that describes the sources of funding for the work included in this submission. Review the submission guidelines for detailed requirements. View published research articles from *PLOS ONE* for specific examples.<br><br>This statement is required for submission and **will appear in the published article** if the submission is accepted. Please make sure it is accurate.<br><br>**Unfunded studies**<br>Enter: *The author(s) received no specific funding for this work.*<br><br>**Funded studies**<br>Enter a statement with the following details:<br>• Initials of the authors who received each award<br>• Grant numbers awarded to each author<br>• The full name of each funder<br>• URL of each funder website<br>• Did the sponsors or funders play any role in the study design, data collection and analysis, decision to publish, or preparation of the manuscript?<br>• **NO** - Include this sentence at the end of your statement: *The funders had no role in study design, data collection and analysis, decision to publish, or preparation of the manuscript.*<br>• **YES** - Specify the role(s) played.<br><br>* typeset	This work was supported by the Economic and Social Research Council (https://esrc.ukri.org) [grant number ES/J500021/1] and the funding was received by SL. The funders had no role in study design, data collection and analysis, decision to publish, or preparation of the manuscript.
**Competing Interests**<br><br>Use the instructions below to enter a competing interest statement for this submission. On behalf of all authors, disclose any competing interests that could be perceived to bias this work—acknowledging all financial support and any other relevant financial or non-financial competing interests.	The authors have declared that no competing interests exist.

Powered by Editorial Manager® and ProduXion Manager® from Aries Systems Corporation

This statement **will appear in the published article** if the submission is accepted. Please make sure it is accurate. View published research articles from *PLOS ONE* for specific examples.<br><br>**NO authors have competing interests**<br><br>Enter: *The authors have declared that no competing interests exist.*<br><br>**Authors with competing interests**<br><br>Enter competing interest details beginning with this statement:<br><br>*I have read the journal's policy and the authors of this manuscript have the following competing interests: [insert competing interests here]*<br><br>* typeset	
**Ethics Statement**<br><br>Enter an ethics statement for this submission. This statement is required if the study involved:<br><br>• Human participants<br>• Human specimens or tissue<br>• Vertebrate animals or cephalopods<br>• Vertebrate embryos or tissues<br>• Field research<br><br>Write "N/A" if the submission does not require an ethics statement.<br><br>General guidance is provided below. Consult the submission guidelines for detailed instructions. **Make sure that all information entered here is included in the Methods section of the manuscript.**	The analysis of secondary data was approved by the London School of Hygiene & Tropical Medicine's Research Ethics Committee (ref: 16190).

Powered by Editorial Manager® and ProduXion Manager® from Aries Systems Corporation

**Format for specific study types**<br><br>**Human Subject Research (involving human participants and/or tissue)**<br>• Give the name of the institutional review board or ethics committee that approved the study<br>• Include the approval number and/or a statement indicating approval of this research<br>• Indicate the form of consent obtained (written/oral) or the reason that consent was not obtained (e.g. the data were analyzed anonymously)<br><br>**Animal Research (involving vertebrate animals, embryos or tissues)**<br>• Provide the name of the Institutional Animal Care and Use Committee (IACUC) or other relevant ethics board that reviewed the study protocol, and indicate whether they approved this research or granted a formal waiver of ethical approval<br>• Include an approval number if one was obtained<br>• If the study involved *non-human primates*, add *additional details* about animal welfare and steps taken to ameliorate suffering<br>• If anesthesia, euthanasia, or any kind of animal sacrifice is part of the study, include briefly which substances and/or methods were applied<br><br>**Field Research**<br><br>Include the following details if this study involves the collection of plant, animal, or other materials from a natural setting:<br>• Field permit number<br>• Name of the institution or relevant body that granted permission	
**Data Availability**<br><br>Authors are required to make all data underlying the findings described fully available, without restriction, and from the time of publication. PLOS allows rare exceptions to address legal and ethical concerns. See the PLOS Data Policy and FAQ for detailed information.	Yes - all data are fully available without restriction

A Data Availability Statement describing where the data can be found is required at submission. Your answers to this question constitute the Data Availability Statement and **will be published in the article**, if accepted.<br><br>**Important:** Stating 'data available on request from the author' is not sufficient. If your data are only available upon request, select 'No' for the first question and explain your exceptional situation in the text box.<br><br>Do the authors confirm that all data underlying the findings described in their manuscript are fully available without restriction?	
**Describe where the data may be found in full sentences. If you are copying our sample text, replace any instances of XXX with the appropriate details.**<br><br>• If the data are **held or will be held in a public repository**, include URLs, accession numbers or DOIs. If this information will only be available after acceptance, indicate this by ticking the box below. For example: *All XXX files are available from the XXX database (accession number(s) XXX, XXX.*).<br>• If the data are all contained **within the manuscript and/or Supporting Information files**, enter the following: *All relevant data are within the manuscript and its Supporting Information files.*<br>• If neither of these applies but you are able to provide **details of access elsewhere**, with or without limitations, please do so. For example:<br><br>*Data cannot be shared publicly because of [XXX]. Data are available from the XXX Institutional Data Access / Ethics Committee (contact via XXX) for researchers who meet the criteria for access to confidential data.*<br><br>*The data underlying the results presented in the study are available from (include the name of the third party*	The National Family Health Survey data used to support the findings of this study have been deposited in the Measure DHS repository http://www.measuredhs.com. The Study on global AGEing and adult health data have been deposited in the WHO-SAGE repository at https://www.who.int/en. The Sample Registration System data have been deposited in the Office of the Registrar General & Census Commissioner repository at http://www.censusindia.gov.in/vital_statistics. The World Population Prospects 2017 data is available online at https://population.un.org/wpp/Download/Standard/Population/ and the World Urbaniztion Prospects 2018 is available at https://population.un.org/wup/Download/.

and contact information or URL).<br>• This text is appropriate if the data are owned by a third party and authors do not have permission to share the data.<br><br>* typeset	
Additional data availability information:	

Manuscript (main)

Click here to download Manuscript Luhar et al PLoS One Sub.docx

Forecasting the Prevalence of Overweight and Obesity in India to 2040

Shammi Luhar[1*], Ian M. Timæus[1,2], Rebecca Jones[3], Solveig Cunningham[4], Shivani A. Patel[4], Sanjay Kinra[1], Lynda Clarke[1], Rein Houben[1]

1 London School of Hygiene & Tropical Medicine, London, UK

2 University of Cape Town, South Africa

3 Laney Graduate School, Emory University, Atlanta GA, USA

4 Hubert Department of Global Health, Emory University, Atlanta GA, USA

* Corresponding author E-mail: shammi.luhar@lshtm.ac.uk

Abstract

*Background***:** In India, the prevalence of overweight and obesity has increased rapidly in recent decades. Given the association between overweight and obesity with many non-communicable diseases, forecasts of the future prevalence of overweight and obesity can help inform policy in a country where around one sixth of the world's population resides.

Methods: We used a system of multi-state life tables to forecast overweight and obesity prevalence among Indians aged 20-69 years by age, sex and urban/rural residence to 2040. We estimated the incidence and initial prevalence of overweight using nationally representative data from the National Family Health Surveys 3 and 4, and the Study on global AGEing and adult health, waves 0 and 1. We forecasted future mortality, using the Lee-Carter model fitted life tables reported by the Sample Registration System, and adjusted the mortality rates for Body Mass Index using relative risks from the literature.

Results: The prevalence of overweight will more than double among Indian adults aged 20-69 years between 2010 and 2040, while the prevalence of obesity will triple. Specifically, the prevalence of overweight and obesity will reach 30.4% (26.5%-34.6%) and 9.6% (5.6%-13.6%) among men, and 27.4% (23.9%-30.9%) and 13.9% (10.0%-17.5%) among women, respectively, by 2040. The largest increases in the prevalence of overweight and obesity between 2010 and 2040 is expected to be in older ages, and we found a larger relative increase in overweight and obesity in rural areas compared to urban areas. The largest relative increase in overweight and obesity prevalence was forecast to occur at older age groups.

Conclusion: The overall prevalence of overweight and obesity is expected to increase considerably in India by 2040, with substantial increases particularly among rural residents and older Indians. Detailed predictions of excess weight are crucial in estimating future non-communicable disease burdens and their economic impact.

Background

Approximately 39% of the global adult population were classified as overweight (Body Mass Index (BMI) 25.0 – 29.9 kg/m^2) or obese (BMI > 29.9kg/m^2) in 2014; a doubling since 1975[1]. Whereas the prevalence of obesity was 6.4% among women and 3.2% among men in 1975, it had risen to 14.9% and 10.8%, respectively by 2014[1]. In developing countries like India, the increasing prevalence of overweight and obesity has coincided with the demographic and epidemiological transitions, in which mortality and fertility have declined, and lifestyle-related diseases have become more common[2–4].

The prevalence of overweight and obesity in India is increasing faster than the world average. For instance, the prevalence of overweight increased from 8.4% to 15.5% among women between 1998 and 2015, and the prevalence of obesity increased from 2.2% to 5.1% over the same period[5–7]. This fast-paced growth has been accompanied by notable increases in the burden of non-communicable diseases (NCDs). Whereas in 1990 the number of life years lost to disability (DALYs) attributable to communicable, maternal, neonatal and nutritional disorders exceeded that attributable to NCDs in virtually all of India's states, currently the opposite is true[3]. Given the extent of the increase in prevalence of overweight and obesity, and its relationships with NCDs[8], reliably predicting its future prevalence has become increasingly important.

Despite this, few studies have attempted to estimate future trends in overweight and obesity in India. One study that reports on global trends estimated that 27.8% of all Indians would be overweight, and 5.0% obese, by 2030[9]. Another study estimated that around 20% of rural Indian adults will be either overweight or obese by 2030[10]. However, these previous studies have merely extrapolated previous trends in prevalence without accounting for a changing population at risk of becoming overweight or obese which declines as the proportion of the population classified as overweight or obese increases.

Simulation models offer a more sophisticated alternative to the extrapolation of secular trends and may produce more accurate forecasts. For example, as an internally logical system, the population at-risk of becoming overweight or obese is regularly updated at each forecasted time interval. Such models therefore

allow the incorporation of the impact on future prevalence of past increases in the incidence of overweight or obesity[11]. Additionally, the logical framework enables the estimation of potential impacts of policy decisions, directed at the incidence of overweight and obesity[11,12], and identification of at-risk subpopulations[11,13,14]. This analysis brings together nationally-representative data from a range of publicly available sources in a dynamic simulation model to forecast the future prevalence of overweight and obesity in India to 2040 among adults aged 20-69 years.

Methods

Data

1. *National Family and Health Survey (NFHS)*

The nationally-representative NFHS collects health and demographic data among women aged 15-49 years and men aged 15-54 years. NFHS 3 (2005-06) interviewed 124,385 and 74,369 adult women and men respectively, and NFHS 4 (2015-16) contains data on 625,000 adult women and 93,065 adult men[6,7].

2. *The Study on global AGEing and adult health (SAGE)*

SAGE Waves 0 (2002-04) and 1 (2007-10) contain longitudinal health and demographic data on people aged 50 or more years from six states which are believed to be nationally-representative [15]. Wave 0 collected health information on 2559 adults aged 50 or more years, whereas Wave 1 collected data on approximately 3000 men and 3000 women aged 50 or more years.

3. *Sample Registration System (SRS)*

The SRS reports sex- and residence-specific abridged life tables by five-year age groups for each state for every year between 1997 and 2015[16–21]. The SRS dually records deaths using representative samples from across the country[22].

4. *United Nations World Population Prospects 2017 and World Urbanization Prospects 2018*

The 2017 round of the World Population Prospects includes population projections and estimates by the Population Division of the Department of Economic and Social Affairs of the United Nations Secretariat[23]. The Division uses the cohort-component method for each country and major geographical region to produce population projections under a number of different future fertility scenarios. Separate urban and rural projections that are consistent with the national projections are reported by the UN World Urbanization Prospects 2018[24].

Model inputs

From these data sources, we extracted the following model inputs for the age, sex and residence-specific forecasts of overweight prevalence in India:

Age-specific prevalence of overweight and obesity

We estimated the prevalence of overweight and obesity among individuals aged 20-49 using the BMI variable in NFHS-3 and NFHS-4, whereby individuals with a BMI>24.9kg/m^2 and <30.0kg/m^2 were classified as overweight, and those with a BMI>29.9kg/m^2 were classified as obese following the World Health Organization's (WHO) recommendations[8]. Pregnant women (5.2% of women in NFHS 3 and 4.4% in NFHS 4) were excluded from our sample as their pregnancy could give misleadingly high BMI scores.

We used survey weights to calculate age-specific prevalence accounting for the complex survey design and based the baseline (2010) age-specific prevalence on the mid-point prevalence of the two surveys. To estimate the baseline prevalence of overweight among individuals aged 50-69, we used the BMI variable, and the same cut-offs, in SAGE wave 1 (2007-10).

Age-specific incidence of overweight and obesity and age-specific rates of urbanization

We used the changing prevalence of overweight and obesity between 2005 and 2015 among the population aged 20-49 years to estimate the age-specific incidence of overweight among the underweight/normal weight population, and incidence of obesity among overweight individuals in our baseline year of 2010. We used the iterative intracohort interpolation procedure[25] whereby the observed changes in overweight status to specific cohorts are translated into age-specific rates for the inter-survey interval (a more detailed explanation is presented in Appendix I). The age-specific rates were estimated separately by sex, residency (urban and rural) and age (20-24, 25-29, 30-34, 35-39, 40-44, 45-49 years).

Age-specific rates of urbanization were also calculated by the same method, using the age-specific proportions of the population in urban and rural areas in the two NFHS surveys.

For those aged 50-69, we calculated the incidence of overweight and obesity using longitudinal data from SAGE waves 0 and 1 for men and women separately by dividing the number of incident cases by the person-years of exposure. As information on the exact time an incident case occurred was not available, incident cases were assumed to have occurred at the halfway-point between the two waves. We calculated an overall incidence rate among men and women separately, and indirectly standardized the rates using the age-distribution of obesity incidence from a study in the United States[26] that used data from the Behavioral Risk Factor Surveillance System in order to obtain net rates for the following age groups: 50-54, 55-59, 60-64, and 65-69 years (Appendix II).

We fitted a spline to smooth the age-specific incidence rates across the lifespan and used age-specific incidence by the five-year age groups in the final analysis.

Remission

We incorporated the potential for individuals to transition from overweight and obesity to lower BMI groups by modelling gross, rather than net incidence rates at all ages. We used rates of remission that allowed our model with gross rates to closely match the measured age-specific prevalence in 2015 from NFHS-4. To estimate remission in older ages, we applied an odds ratio of remission in older ages (50+ years), relative to younger ages. A prospective study in rural India carried out between 2008 and 2017[27] found an elevated odds of remission from higher to lower BMIs of 1.74 and 2.12 among older aged men and women, relative to younger counterparts.

Current and future age-, sex- and urban/rural residence- specific mortality rates

We converted conditional mortality probabilities reported by the SRS to age-specific mortality rates from 1997 to 2013 using standard demographic procedures[28] and used these rates to forecast future mortality to 2040 using the Lee-Carter method[29,30]. In brief, the Lee-Carter method summarizes a series of sets of age-specific mortality rates for successive periods of time by its average age-schedule, age-specific deviations from the average age-schedule, and the trend in the overall level of mortality over time. The forecast is contingent on the extrapolation of this latter parameter (Appendix III).

Relative risk of dying for those overweight or obese compared with those who are not

We adjusted the forecasted mortality rates to account for differential mortality between overweight and obese individuals and those who are in lower weight categories. Relative risks of dying, based on BMI group, were adopted from the findings reported in a study that examined the association between BMI and mortality in Mumbai[31]. This study reported relative risks of dying for those who are overweight excluding obesity (OW), relative to normal (*N*) weight, those who are obese (*OB*) relative to normal weight, and those who are underweight (*UW*) relative to normal weight. The authors report risk ratios, along with confidence intervals, for men and women aged 35-59 and 60 or more, separately. As the study did not calculate risk ratios for individuals aged 20-34, we assumed that the relative risk of dying at 35-59 also prevailed at these ages. We obtained separate relative risks of dying for those who are overweight and obese relative to new reference categories of ‘not overweight’ and ‘not obese’ using a basic algebraic approach (Appendix IV), and subsequently used these relative risks to calculate BMI specific rates of mortality using the population level mortality rate. Below we present an example of the calculation of obesity-specific mortality rates.

$$m_{a,t}^{Not\ OB} = \frac{m_{a,t}}{(\delta_{a,t}^{OB} * R_a) - \delta_{a,t}^{OB} + 1}$$

$$m_{a,t}^{OB} = m_{a,t}^{Not\ OB} * R_a$$

Where $\delta_{x,t}^{OB}$ refers to the prevalence of obesity in age group *a* at time *t*, and R_a is the relative risk of dying among obese adults relative to non-obese counterparts aged *a*.

Age-, sex- and urban/rural residence-specific population in 2010

Estimates of the 2010 urban and rural population were taken from the *World Urbanization Prospects*[24] and disaggregated using the average age-group and sex structure of urban and rural populations separately, which we obtained from the NFHS-3 and NFHS-4[6,7].

Population aged 20-24 entering the simulation at every interval

The new entrants aged 20-24 that join the urban and rural populations in each time interval were estimated using the projected population aged 20-24 from the medium fertility projection scenario by the United Nations' *World Population Prospects*[23] and split into the projected proportions of the population in urban and rural areas from the *World Urbanization Prospects*[24].

The Model

We estimated the future prevalence of overweight and obesity through 2040 using an age-stratified simulation model based on a system of multi-state lifetables[32], that moved individuals through mutually-exclusive health states depending on our estimated transition rates as they age. The model operated in discrete time, estimating the prevalence of overweight and obesity separately among men and women in urban and rural areas separately, at five-yearly intervals between 2010 and 2040. The system of multi-state lifetables is shown in Figure 1.

Figure 1. Compartmental model of forecasted overweight and obesity prevalence in India

[FIGURE 1 ABOUT HERE]

Most epidemiological studies apply transition probabilities to the population at risk of a transition at the beginning of a time period to determine the distribution of the population across health states in a succeeding time period, without taking account of a changing population at risk within a time interval. This is due to individuals being able to enter and re-enter a particular BMI group within a time interval. In order to sufficiently account for this, we employed a multi-state lifetable system first developed by Schoen and Nelson (1974) who addressed questions about flows in and out of marriage in the UK and USA. Rather than work with transition probabilities derived from the rates, this approach to forecasting health states uses the rates directly. A detailed description of this method is included in Appendix V.

Assumptions

Firstly, between 2005 and 2015 the pace of increase in prevalence of overweight and obesity is faster in rural populations than in urban areas. As the prevalence of overweight and obesity among the 20-24 population is not determined within the model, we assumed that the rate of increase in this age group observed from the overweight and obesity prevalence in the NFHS data decreased and converged towards a 0% increase by 2040, so as not to overinflate our estimates. Additionally, our baseline forecasts, assumed that the empirically estimated overweight and obesity incidence for each demographic group for the baseline year (2010) applied throughout the forecasting period. This assumption provides a clear and easily interpretable counterfactual scenario against which to compare other scenarios whereby incidence is allowed to vary over the forecast period. For simplicity, we assumed that there is no migration in and out of India.

Finally, it was assumed that the rate of urbanisation measured between 2005 and 2015 prevailed throughout the forecast period.

Uncertainty Analyses

To obtain uncertainty bounds for our estimates we simultaneously selected random prevalence, incidence and mortality rates from the distributions that informed their uncertainty. We repeated the simulation 5000 times and we reported the median estimate as the final point estimate, whilst the range of estimates for each population subgroup informed our uncertainty bounds. The analysis was conducted in R version 3.5.1.

Sensitivity Analyses

The future incidence of overweight may continue to increase due to economic development creating an increasingly obesogenic environment. To explore the implications of this potential trend, we included additional scenarios. Scenario 1 involved examining the effect on future prevalence the incidence parameter increasing at a constant annual rate of 1%. In Scenario 2, we examined the effect on future overweight and obesity of the urbanization rate being set at its upper confidence bound throughout the forecast period. Finally, Scenario 3 examined the extent to which the total prevalence of overweight and obesity prevalence would change if no further urbanization were to take place to 2040. Although unrealistic, this provides an understanding as to the extent to which the future increase in prevalence is driven by future urbanization.

Results

Nationally, our model estimates that the prevalence of overweight among women will increase from 14.7% (13.6%-15.8%) to 27.3% (23.9%-30.9%) between 2010 and 2040, whereas the prevalence of obesity is forecasted to increase from 4.4% (3.9%-4.9%) to 14.0% (10.0%-17.5%) over the same period (Figure 2).

Among men, the prevalence of overweight and obesity is forecasted to increase from 12.6% (11.4%-13.8%) and 2.4% (2.0%-2.9%) in 2010 to 30.4% (26.5%-34.6%) and 9.6% (5.6%-13.6%), respectively, by 2040 (Figure 2).

The prevalence of overweight and obesity is forecasted to remain higher in urban areas, compared with rural areas, reaching 32.1% (27.3%-38.0%) and 19.6% (13.8%-24.8%), respectively among urban women and 37.1% (31.1%-44.4%) and 11.4% (5.4%-17.0%), respectively, among urban men by 2040. However, the relative increase will be larger in rural areas, where the baseline model forecasts that the prevalence of obesity among women will be 4 times higher in 2040 than in 2010 in rural areas, compared to a 2.6 times higher prevalence of obesity in urban areas over the same period.

The model also predicts larger increases in the prevalence of overweight and obesity in older age groups. Using the broad age groups in Tables 1 and 2, we find that, for example, among men, the prevalence of overweight in urban areas among 55-69-year-olds is predicted to almost quadruple from 10.7% (8.1%-13.5%) to 38.6% (30.7%-47.8%) between 2010 and 2040, whereas the prevalence of overweight in rural areas is predicted to increase from 4.7% (3.4%-6.0%) to 30.2% (23.0-37.7%). On the other hand, our model predicts that younger age groups in our model will experience the smallest absolute increase in the overweight (Figures 3 and 4).

Under the assumption of a 1% annual increase in incidence of overweight and obesity from 2015, we expect the national prevalence of overweight to increase to 29.7% (26.1%-33.9%) by 2040 among women and to 33.2% (29.4%-37.6%) over the same period for men (Figure 6). Over the same period, we expect the national prevalence of obesity to increase to 16.9% (12.2%-21.4%) among women and 12.2% (7.1%-17.0%) among men. Under the high urbanization scenario, we find that the future national prevalence of overweight between 2010 and 2040 will increase to 31.7% (27.4%-35.8%) among women, compared to 25.0% (23.5%-30.9%) under an assumption of no further urbanization. In contrast, although the high urbanization scenario for men finds a 1.5% higher prevalence of overiwght among men in 2040, compared to the scenario of no

further urbanization, the prevalence of obesity in 2040 does not vary notably between these scenarios (Figure 5).

Figure 2. Forecasted prevalence of overweight and obesity at ages (20-69 years) 2010–2040

[FIGURE 2 ABOUT HERE]

Figure 3. Forecasted age-specific prevalence of overweight and obesity to 2040 (men)

[FIGURE 3 ABOUT HERE]

Figure 4. Forecasted age-specific prevalence of overweight and obesity to 2040 (women)

[FIGURE 4 ABOUT HERE]

Table 1. Forecast percentage of overweight and obese in the population to 2010–2040 (men)

Weight	***Residence***	***Year***	***20-34***			***35-54***			***55-69***			***All***		
			Point est.	***Lower***	***Upper***	***Point est.***	***Lower***	***Upper***	***Point est.***	***Lower***	***Upper***	***Point est.***	***Lower***	***Upper***
Overweight	Rural	2010	8.4	6.6	10.2	12.3	9.9	14.6	4.7	3.4	6.0	9.2	7.9	10.6
		2020	14.5	11.4	18.0	21.7	17.5	26.5	18.3	14.1	22.4	18.0	14.5	21.7
		2030	17.6	13.8	22.1	25.9	19.8	32.3	26.5	20.2	33.0	22.8	17.5	28.2
		2040	19.0	14.9	24.0	27.9	20.8	35.4	30.2	23.0	37.7	25.4	19.3	32.0
Obese	Rural	2010	1.2	0.7	1.7	2.3	1.6	3.1	0.8	0.2	1.3	1.6	1.1	2.0
		2020	2.6	1.3	3.9	5.4	3.5	7.6	3.1	1.7	4.7	3.8	2.3	5.2
		2030	3.7	1.9	5.6	8.5	4.8	12.3	6.4	3.4	9.9	6.2	3.4	8.9
		2040	4.3	2.1	6.5	10.6	5.9	15.3	9.4	4.7	14.1	8.2	4.4	11.9
Overweight	Urban	2010	15.9	13.9	17.9	25.8	22.7	28.8	10.9	8.1	13.5	19.0	17.1	20.8
		2020	24.6	21.2	28.5	38.4	33.0	44.0	30.8	25.2	36.4	31.5	27.2	36.0
		2030	28.3	24.3	32.9	41.6	34.6	49.0	37.4	30.3	45.4	35.8	30.2	42.2
		2040	30.2	25.8	35.1	42.2	35.3	50.4	38.6	30.7	47.8	37.1	31.1	44.4
Obese	Urban	2010	3.2	2.2	4.3	5.7	4.3	6.9	1.7	0.6	2.9	4.0	3.1	4.8
		2020	5.2	3.3	7.2	8.3	4.8	11.8	6.2	2.5	9.6	6.7	4.0	9.3
		2030	7.0	4.4	9.7	11.2	5.5	16.8	10.2	3.3	17.1	9.4	4.9	13.9
		2040	8.0	5.0	11.0	13.5	6.6	20.4	12.9	3.4	21.8	11.4	5.5	17.0

Table 2. Forecast percentage of overweight and obese in the population to 2010–2040 (women)

Weight	***Residence***	***Year***	***20-34***			***35-54***			***55-69***			***All***		
			Point est.	***Lower***	***Upper***	***Point est.***	***Lower***	***Upper***	***Point est.***	***Lower***	***Upper***	***Point est.***	***Lower***	***Upper***
Overweight	Rural	2010	8.0	6.5	9.6	14.9	12.8	17.0	9.3	7.7	10.8	10.9	9.7	12.3
		2020	12.6	10.2	15.1	21.5	17.4	25.8	21.9	17.4	26.9	18.1	14.9	21.6
		2030	14.7	11.8	17.6	24.6	19.4	30.2	27.7	21.2	35.9	21.9	17.3	26.8
		2040	15.8	12.7	18.9	25.9	20.4	32.1	30.2	22.5	39.6	24.0	18.8	29.9
Obese	Rural	2010	1.7	1.2	2.2	3.9	2.8	4.9	2.5	1.5	3.6	2.7	2.1	3.3
		2020	3.3	2.3	4.2	7.1	5.1	9.1	5.6	2.9	8.4	5.3	3.9	6.7
		2030	4.4	3.0	5.7	9.9	7.1	13.0	9.6	5.0	14.4	7.9	5.5	10.5
		2040	5.0	3.4	6.5	11.8	8.4	15.5	12.4	6.4	18.4	9.9	6.6	13.3
Overweight	Urban	2010	17.4	15.7	19.1	30.6	28.8	32.6	21.3	17.6	25.0	23.2	21.9	25.0
		2020	21.5	19.1	23.8	35.0	30.8	40.1	32.5	25.4	41.2	29.1	25.2	33.6
		2030	23.9	21.2	26.7	35.7	31.1	41.4	34.7	27.0	46.0	31.2	26.7	36.8
		2040	25.2	22.3	28.1	35.9	31.2	41.6	35.0	27.3	46.7	32.1	27.3	38.0
Obese	Urban	2010	5.4	4.5	6.3	12.3	10.5	13.9	5.9	3.7	8.1	8.2	7.1	9.2
		2020	7.2	5.9	8.5	18.8	14.7	22.6	16.0	8.3	22.8	13.6	10.3	16.8
		2030	8.9	7.2	10.6	21.7	16.6	26.4	24.0	12.8	33.1	17.5	12.5	21.9
		2040	10.0	8.1	11.9	23.5	17.9	28.7	26.5	14.0	36.5	19.7	13.8	24.8

Figure 5. Forecasted prevalence overweight and obesity to 2040 under the four different scenarios tested

[FIGURE 5 ABOUT HERE]

Discussion

Overall, we predict that the prevalence of overweight will increase approximately double among Indian adults aged 20-69 years between 2010 and 2040, whilst the prevalence of obesity is expected to increase approximately three-fold over the same period. Specifically, amongst men, we predict that the prevalence of overweight and obesity respectively will reach around 30% and 10%, whilst 27% and 14% of women are expected to be overweight and obese, respectively, by 2040. Our model additionally predicts an ageing distribution of overweight and obesity, with the largest relative increases in prevalence observed among the 55-69-year age group (in this age group the prevalence of obesity among women is predicted to increase almost 6-fold in rural areas and almost 5-fold in urban areas over the forecast period). Whilst prevalence of overweight and obesity is expected to be higher in urban areas throughout the forecast period, we predict larger relative increases in their prevalence in rural areas.

Our forecasting model has a number of limitations. Firstly, we determine the future prevalence of the new cohorts of 20-24-year individuals outside of the model, where we applied a declining rate of increase in prevalence, so as to not grossly inflate future prevalence in this age group to unrealistic levels. Studies have documented increasing overweight prevalence among young adults in India, especially among men and high socioeconomic status individuals[33].

Secondly, we used standard global BMI thresholds over which there is some controversy. Some researchers advocate for using lower BMI thresholds for South Asians[34] due to a higher percentage of body fat among South Asians compared to Caucasians of the same BMI[35,36]. As research has documented a nearly 10-15%

higher prevalence of overweight among individuals with Asian heritage if Asian-specific cut-offs are used[36], we may have underestimated our final predictions. Others have found no higher risk of mortality among obese Asians compared to obese non-Asians, and advocate for global consistency in the definition of overweight and obesity[9,37,38]. We opted to use global cut-offs for this reason and in order to facilitate direct comparison of the predictions with similar forecasting studies in Western countries[39–41].

Thirdly, our assumption of no migration in and out of India may slightly bias our findings if individuals leaving India to elsewhere are more likely to be overweight or obese than individuals who remain or enter. Any bias attributable to our assumption of zero migration in an out of India is however likely to be negligible as the number of annual net migrants (minus 2.5 million in 2017 according to the World Bank[42]) currently represents less than 1% of the total population[23].

Despite these limitations, our study has a number of strengths. Firstly, we have quantified the future prevalence of overweight and obesity in India using the most recent nationally-representative publicly available data. Our model is able to reflect the changing demographic profile of India in future estimates of the prevalence of overweight and obesity and, in addition, to incorporate future rates of urbanization. Additionally, it models the future age- and sex-specific prevalence of overweight as a function of past and current age- and sex-specific incidence and mortality; reflecting the real-life lag between demographic changes, changes in incidence and mortality and their effect on the overall prevalence at various ages in the future. Unlike previous studies predicting future prevalence of overweight and obesity in India[9,10], we forecasted prevalence for age-stratified subgroups as well as generating aggregated forecasts, putting emphasis on demographic groups that are expected to experience particularly high increases in prevalence.

Few studies have attempted to forecast the future prevalence of overweight in India. One study from 2005 predicted that the prevalence of overweight among Indian adults, assuming a continuation of past trends, will increase to 27.8% by 2030, whist the prevalence of obesity is predicted to reach 5.0%[9]. The overweight estimations closely resemble our predictions for men and are slightly above what we predict for women.

However, our model predicts a considerably higher prevalence of obesity by 2030, with 11.5% of women and 7.4% of men predicted to be obese by 2030.

Another study, focusing on rural India estimated that the prevalence of overweight will approach 20% among men and just exceed 20% among women aged 18 and over by 2030[10]. We, on the other hand, expect 28.9% of men and 29.8% of women to be either overweight or obese by 2030. The discrepancy between these two separate findings may indeed be due to the different methodologies adopted but is more likely explained the fact that our study included older age groups among whom overweight and obesity prevalence is expected to increase most substantially by 2030.

The differences between our results and previous forecasts of the prevalence of overweight in India may also be explained by our attempts to take into account some of the heterogeneity in the incidence of overweight an obesity and mortality sub-nationally, estimating urban and rural outcomes separately for men and women. Also, instead of making *a priori* assumptions about the future prevalence of overweight and obesity, for instance a linearly increasing prevalence rate, we model future prevalence as a function of a continuously updated 'population at-risk'. Although we expect our baseline results to be relatively conservative, as we fix age-specific incidence rates over the forecast period, we expect them to be more accurate than previous attempts. This is due to our use of the most up-to-date data, and the fundamental differences in modelling approaches.

The ageing age distribution of overweight prevalence is likely to be driven by a cohort effect. Previous research has reported a peak in the prevalence of overweight in the 40-49 age group in 2005 in India, whereas in more economically developed countries, the prevalence in the same year peaks in the 60-69 age group[9]. Our finding of an older age distribution of both overweight and obesity prevalence in 2040, compared with 2010, may be associated with India's increasing resemblance to higher-income countries in terms of overall prevalence of overweight and economic development. When we tested our forecasts holding future mortality rates at the 2010 level, future prevalence did not notably differ from the forecasts in which future mortality

was allowed to decline. Consequently, previous and continuing increases in longevity are not likely to be an important driver of this ageing age distribution of overweight.

We confirmed that our model predictions were very similar to the 2015 age-specific prevalence estimates reported by the NFHS. Another way we assessed the ability of our model to accurately predict future overweight was to compare our output with collected data on overweight prevalence from a data source that was not used in the parameterization of our model. The National Nutrition Monitoring Bureau (NNMB) reports that in 2017 the prevalence of overweight in urban areas was 34% among men. In our model, the prevalence of overweight and obesity combined among urban men is 35%, and the NNMB estimate falls comfortably within our uncertainty bound of 29.3% - 40.9%. The NNMB also reports a point estimate of 44.0% prevalence among urban women in 2017, falling within our uncertainty bound of 34.3% – 46.8%, although our point estimate is lower, at 40.4%. We would expect the interval around their estimate to considerably overlap with ours, however this interval was unavailable. Although NNMB estimates fall comfortably within our uncertainty bound, differences between point estimates can derive from a number of sources. Firstly, different sampling frames are used in the surveys, whereby the NNMB in urban areas selects a sampling frame from under half (16) of Indian states they believe to accurately reflect national trends[43]. Additionally, the NNMB included individuals aged 70 years or more, the majority of whom are likely to be urban women due to their higher life expectancy[21].

Our baseline findings, in which incidence of overweight and obesity remains constant over the forecast period, suggest that the future prevalence of obesity is likely to be lower than that currently observed in some of the world's most industrialized economies. For instance, recent a recent survey has found that using the same BMI cut-offs as in our study, 40.4% and 35.0% of women and men in the US, respectively were classified as obese in 2013-14[44], whereas we find that in urban areas, the relatively more obesogenic environment, 19% of women and 16% of men are likely to be obese by 2040, however, this is one of our most conservative estimates, assuming a constant rate of incidence over the forecast period. Nevertheless, a 1% annual increase in incidence, corresponding to a 35% overall increase in incidence over the forecast

period only leads to a 2% higher prevalence in overweight and obesity by 2040, suggesting that much of the future forecasted prevalence will be determined by the changing demographic profile and background BMI trends of India. Attempts to reduce the forecasted prevalence in 2040 may therefore aim to target a reduction in overweight and obesity incidence, starting among children and adolescents yet to pass through the 20-69-year-old population.

The future task of tackling the increasing disease burden associated with the tripling of obesity prevalence will be particularly challenging in India, given its already high burden of infectious diseases[3], and given that it is expected to have the largest population in the world by 2024[23]. Obesity is the main risk factor for a range of NCDs, including diabetes. A meta-analysis of prospective cohort studies found a 7.19 times higher risk of diabetes among obese individuals compared to normal weight individuals[45]. Given that diabetic individuals are more at risk of diabetes related complications, including long-term vascular complications affecting the kidneys, heart, and nerves[46], addressing the growing obesity prevalence and ageing pattern of prevalence, is of great urgency.

Although the overall prevalence of overweight is expected to be higher in urban areas, our baseline scenario suggests that in urban India future overweight prevalence may begin to plateau during the forecasting period if incidence remains at 2010 levels over the forecast period, while rural areas will continue to experience an increasing prevalence. On the other hand, our model has predicted an almost linear growth in the prevalence of obesity in both urban and rural areas. Irrespective of future incidence or urbanization rate however, our results suggest that a considerably larger proportion of the population in both urban and rural areas will be either overweight or obese by 2040 compared to 2010, driven by the ageing of overweight and obese younger people and increasing prevalence of overweight and obesity in younger ages.

Close monitoring of these populations may be warranted, and interventions to reduce the overall growth in prevalence way wish to target these populations, particularly among populations susceptible to becoming obese for whom the risk of NCDs is substantially higher[47]. Additionally, health policy planners may wish

to pay particular attention to individuals at younger ages to avoid early onset of overweight and the accumulation of overweight prevalence in older age groups.

Given the considerable heterogeneity in customs, diet and economic development between India's states, these forecasts are likely to mask subnational variation. In future work, an examination of how these forecasts may differ at the state level may be particularly useful for health policy planning as the constitution of India devolves the deliverance of health and nutrition policy to the state level[48].

Our model is simple enough to apply to other developing countries with similarly limited data, and its flexibility can be demonstrated by appropriately adjusting the transition rates[49]. Our predictions can also provide the basis of future modelling studies aiming to quantify both monetary costs and future disease burden associated with excess weight in India[50–54].

Our model predicts a considerable increase and an ageing cohort pattern in overweight and obesity across India to 2040, which could have serious implications for future levels of obesity-related diseases, such as diabetes. Initiatives, such as the integrated National Health Mission[55], which aims to raise overall population health, may wish to use these forecasts to target sub-populations in which the prevalence of excess weight is likely to be highest in the future. Our findings can be extended to quantify the impact of reductions in the incidence of overweight and obesity among certain subgroups and ages. This information may be crucial in estimating the future burden of NCDs, as well as their economic impact.

Data Availability

The National Family Health Survey data used to support the findings of this study have been deposited in the Measure DHS repository http://www.measuredhs.com. The Study on global AGEing and adult health data have been deposited in the WHO-SAGE repository at https://www.who.int/en. The Sample

Registration System data have been deposited in the Office of the Registrar General & Census Commissioner repository at www.censusindia.gov.in.

Conflicts of interest

The authors declare that they have no conflicts of interest

Funding Statement

This work was supported by the Economic and Social Research Council [grant number ES/J500021/1].

Author's contributions

SL conceptualised the study, extracted the relevant data, conducted the analysis and wrote the first draft of the manuscript. RH and IT both conceptualised the study along with SL, helped to develop the methods, and participated in the analysis of the data. RJ, RH, SC, SP, IT, SK, and LC all contributed to the interpretation of the data, contributed to the discussion section, and helped to critically revise the manuscript.

Ethics Statement

The analysis of secondary data was approved by the London School of Hygiene & Tropical Medicine's Research Ethics Committee (ref: 16190).

References

1. Di Cesare, M. *et al.* Trends in adult body-mass index in 200 countries from 1975 to 2014: A pooled analysis of 1698 population-based measurement studies with 19.2 million participants. *Lancet* **387**, 1377–1396 (2016).

2. Tandon, N. *et al.* The increasing burden of diabetes and variations among the states of India: the Global Burden of Disease Study 1990–2016. *Lancet Glob. Heal.* **6**, e1352-62 (2018).

3. Dandona, L. *et al.* Nations within a nation: variations in epidemiological transition across the states of India, 1990–2016 in the Global Burden of Disease Study. *Lancet* **390**, 2437–60 (2017).

4. Prabhakaran, D. *et al.* The changing patterns of cardiovascular diseases and their risk factors in the states of India: the Global Burden of Disease Study 1990–2016. *Lancet Glob. Heal.* **6**, e1339-51 (2018).

5. International Institute for Population Sciences. *National Family Health Survey (NFHS-2), India, 1998-99*. (2000).

6. International Institute for Population Sciences. *National Family Health Survey (NFHS-3), India, 2005-06*. *International Institute for Population Sciences* (2009).

7. International Institute for Population Sciences. *National Family Health Survey (NFHS-4) 2015-16 India*. *International Institute for Population Sciences (IIPS) and ICF* (2017).

8. WHO. Obesity and overweight: Fact sheet. *WHO Media Centre* (2016).

9. Kelly, T., Yang, W., Chen, C.-S., Reynolds, K. & He, J. Global burden of obesity in 2005 and projections to 2030. *Int. J. Obes.* **32**, 1431–1437 (2008).

10. Swain, S. & Chowdhury, S. Trends of nutritional status among rural adults in six states of India: findings from national survey data. *Clin. Epidemiol. Glob. Heal.* **6**, 181–187 (2018).

11. Levy, D. T. & Marsh, T. Simulation Models of Obesity: A Review of the Literature and Implications for

Research and Policy David. *Obes. Rev.* **12**, 378–394 (2015).

12. Carter, R. *et al.* Priority setting in health: Origins, description and application of the Australian Assessing Cost-Effectiveness initiative. *Expert Rev. Pharmacoeconomics Outcomes Res.* **8**, 593–617 (2008).

13. Soneji, S. & King, G. Statistical Security for Social Security. *Demography* **49**, 1037–60 (2012).

14. Preston, S. H., Stokes, A., Mehta, N. K. & Cao, B. Projecting the Effect of Changes in Smoking and Obesity on Future Life Expectancy in the United States. *Demography* **51**, 27–49 (2014).

15. Arokiasamy, P., Parasuraman, S., Sekher, T. V. & Lhungdim, H. *Study on global AGEing and adult health (SAGE), Wave 1*. *World Health Organization* (2013). doi:10.3886/ICPSR28502.v2

16. RGI. *SRS based abridged life tables 1996-2000 and 1997- 2001. SRS Analytical Studies Report No. 2 of 2004*. (2004).

17. RGI. *SRS based abridged life tables 2000-2004. SRS Analytical Studies Report No. 1 of 2007*. (2007).

18. RGI. *SRS Based Abridged Life Tables 2002-2006*. (2008).

19. ORG. *SRS Based Abridged Life Tables 2003-07 to 2006-10*. (2015).

20. ORG. *SRS Based Abridged Life Tables 2010-14.* (2016).

21. ORG. *SRS Based Abridged Life Tables 2011-15*. (2017).

22. ORG. *Office of The Registrar General & Census Commissioner, India. 2011 Sample Registration*. (2011).

23. ONU. *World Population Prospects The 2017 revision*. *World Population Prospects The 2017 Revision* (2017). doi:10.1017/CBO9781107415324.004

24. UN-DESA. World Urbanization Prospects: The 2018 Revision. *Dep. Econ. Soc. Aff.* (2018).

25. Stupp, P. W. Estimating Intercensal Age Schedules by Intracohort Interpolation. *Popul. Index* **54**, 209–24 (1988).

26. Pan, L., Freedman, D. S., Gillespie, C., Park, S. & Sherry, B. Incidences of obesity and extreme obesity among US adults: Findings from the 2009 Behavioral Risk Factor Surveillance System. *Popul. Health Metr.* **9**, 56 (2011).

27. Rai, R. K. *et al.* Prospective cohort study of overweight and obesity among rural Indian adults: Sociodemographic predictors of prevalence, incidence and remission. *BMJ Open* **8**, 1–15 (2018).

28. Preston, S. H., Heuveline, P. & Guillot, M. *Demography, Measuring and Modeling Population Processes*. *Population* (2000). doi:10.2307/1535065

29. Lee, R. D. & Carter, L. R. Modeling and forecasting U.S. mortality. *J. Am. Stat. Assoc.* **87**, 659–71 (1992).

30. Lee, R. & Miller, T. Assessing the performance of the Lee-Carter approach to modeling and forecasting mortality. in *Annual Meeting of the Population Association of America* (2000).

31. Pednekar, M. S., Hakama, M., Hebert, J. R. & Gupta, P. C. Association of body mass index with all-cause and cause-specific mortality: Findings from a prospective cohort study in Mumbai (Bombay), India. *Int. J. Epidemiol.* **37**, 524–35 (2008).

32. Schoen, R. & Nelson, V. Marriage, divorce, and mortality: a life table analysis. *Demography* **11**, 267–290 (1974).

33. Gupta, D. K. *et al.* Secular trends in prevalence of overweight and obesity from 2006 to 2009 in Urban Asian Indian adolescents aged 14-17 years. *PLoS One* **6**, e17221 (2011).

34. Nishida, C. *et al.* Appropriate body-mass index for Asian populations and its implications for policy and intervention strategies. *Lancet* **363**, 157 (2004).

35. Misra, A. Ethnic-Specific Criteria for Classification of Body Mass Index: A Perspective for Asian Indians and American Diabetes Association Position Statement. *Diabetes Technol. Ther.* **17**, 667–71 (2015).

36. Stegenga, H., Haines, A., Jones, K. & Professor, J. W. Identification, assessment, and management of overweight and obesity: Summary of updated NICE guidance. *BMJ* **349**, g6608 (2014).

37. Gu, D. *et al.* Body weight and mortality among men and women in China. *J. Am. Med. Assoc.* **295**, 776–83 (2006).

38. Jee, S. H. *et al.* Body-Mass Index and Mortality in Korean Men and Women. *N. Engl. J. Med.* **355**, 779–87 (2006).

39. Basu, A. Forecasting Distribution of Body Mass Index in United States: Is there more room for growth? *Med. Decis. Mak.* **30**, E1–E11 (2010).

40. Haby, M. M., Markwick, A., Peeters, A., Shaw, J. & Vos, T. Future predictions of body mass index and overweight prevalence in Australia, 2005-2025. *Health Promot. Int.* **27**, 250–260 (2012).

41. Finkelstein, E. A. *et al.* Obesity and severe obesity forecasts through 2030. *Am. J. Prev. Med.* **42**, 563–570 (2012).

42. Data.worldbank.org. Net migration. (2018).

43. NNMB. NATIONAL NUTRITION MONITORING BUREAU (NNMB) Technical Report No. 27. Diet and nutritional status of urban population in India and Prevalence of Obesity, Hypertension, Diabetes and Hyperlipidaemia in Urban men and women. *NATIONAL NUTRITION MONITORING BUREAU (NNMB) Technical Report No. 25* (2017).

44. Flegal, K. M., Kruszon-Moran, D., Carroll, M. D., Fryar, C. D. & Ogden, C. L. Trends in obesity among adults in the United States, 2005 to 2014. *JAMA - Journal of the American Medical Association* **315**, 2284–91 (2016).

45. Abdullah, A., Peeters, A., de Courten, M. & Stoelwinder, J. The magnitude of association between overweight and obesity and the risk of diabetes: A meta-analysis of prospective cohort studies. *Diabetes Res. Clin. Pract.* **89**, 309–319 (2010).

46. Pradeepa, R. & Mohan, V. Prevalence of type 2 diabetes and its complications in India and economic costs to the nation. *Eur. J. Clin. Nutr.* **71**, 816–824 (2017).

47. Ganz, M. L. *et al.* The association of body mass index with the risk of type 2 diabetes: A case-control study nested in an electronic health records system in the United States. *Diabetol. Metab. Syndr.* **6**, 50 (2014).

48. The Constitution of India. (1949).

49. Homer, J., Milstein, B., Dietz, W., Buchner, D. & Majestic, E. Obesity Population Dynamics : Exploring Historical Growth and Plausible Futures in the U . S . in *24th International Systems Dynamics Conference* 29 (2006).

50. Al-Quwaidhi, A. J., Pearce, M. S., Sobngwi, E., Critchley, J. A. & O'Flaherty, M. Comparison of type 2 diabetes prevalence estimates in Saudi Arabia from a validated Markov model against the International Diabetes Federation and other modelling studies. *Diabetes Res. Clin. Pract.* **103**, 496–503 (2014).

51. Al Ali, R. *et al.* Forecasting future prevalence of type 2 diabetes mellitus in Syria. *BMC Public Health* **13**, 507 (2013).

52. Abu-Rmeileh, N. M. E., Husseini, A., Capewell, S. & O'Flaherty, M. Preventing type 2 diabetes among Palestinians: Comparing five future policy scenarios. *BMJ Open* **3**, e003558 (2013).

53. Sözmen, K., Unal, B., Capewell, S., Critchley, J. & O'Flaherty, M. Estimating diabetes prevalence in Turkey in 2025 with and without possible interventions to reduce obesity and smoking prevalence, using a modelling approach. *Int. J. Public Health* **60**, 13–21 (2014).

54. Saidi, O. *et al.* Forecasting Tunisian type 2 diabetes prevalence to 2027: Validation of a simple model. *BMC Public Health* **15**, 104 (2015).

55. D'Silva, J. Can India pull off its ambitious National Health Mission? *BMJ* **346**, f2134 (2013).

Figure 1

Click here to download Figure Figure1.tiff

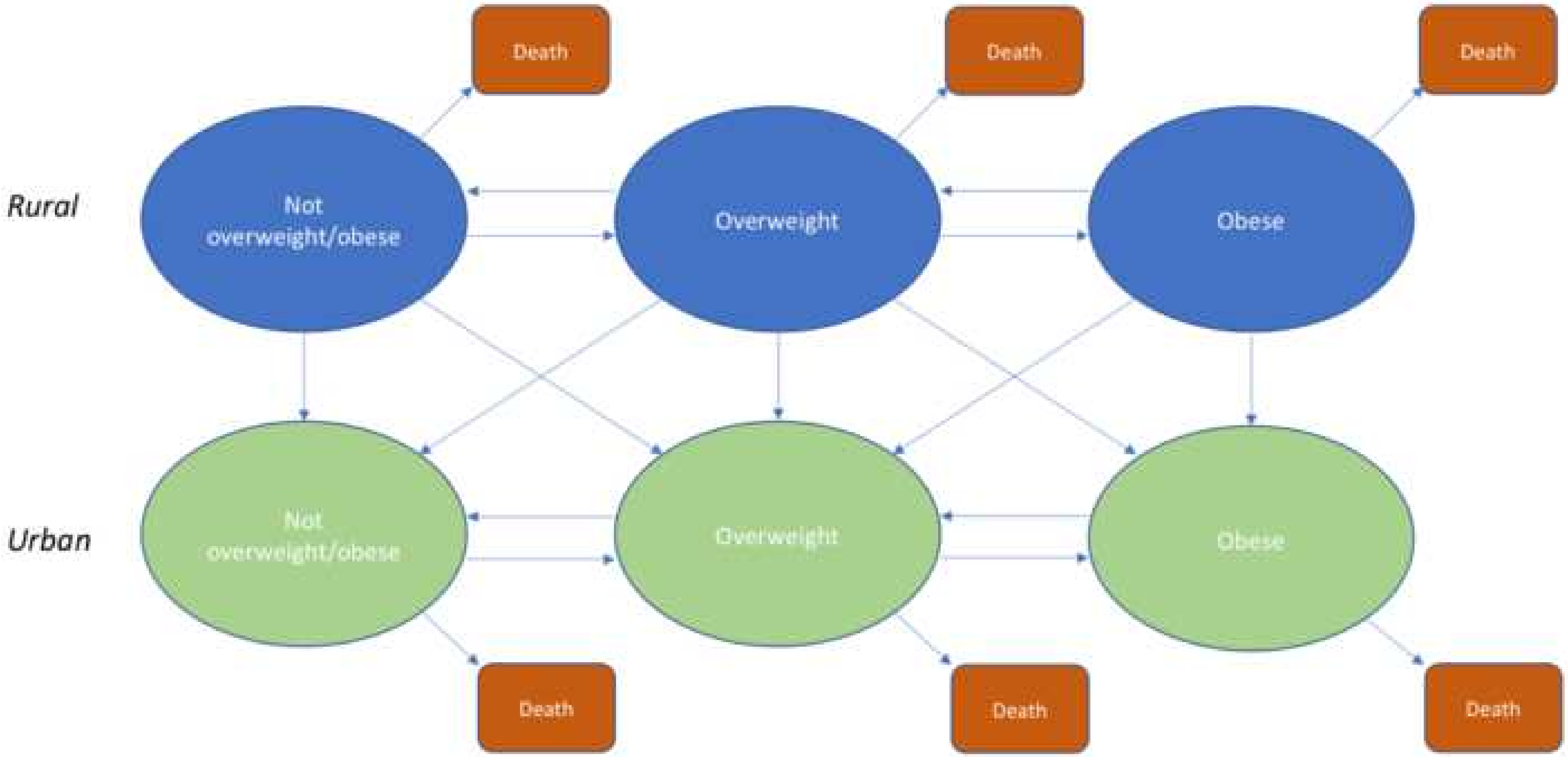

Figure 2

Click here to download Figure Figure 2.tiff

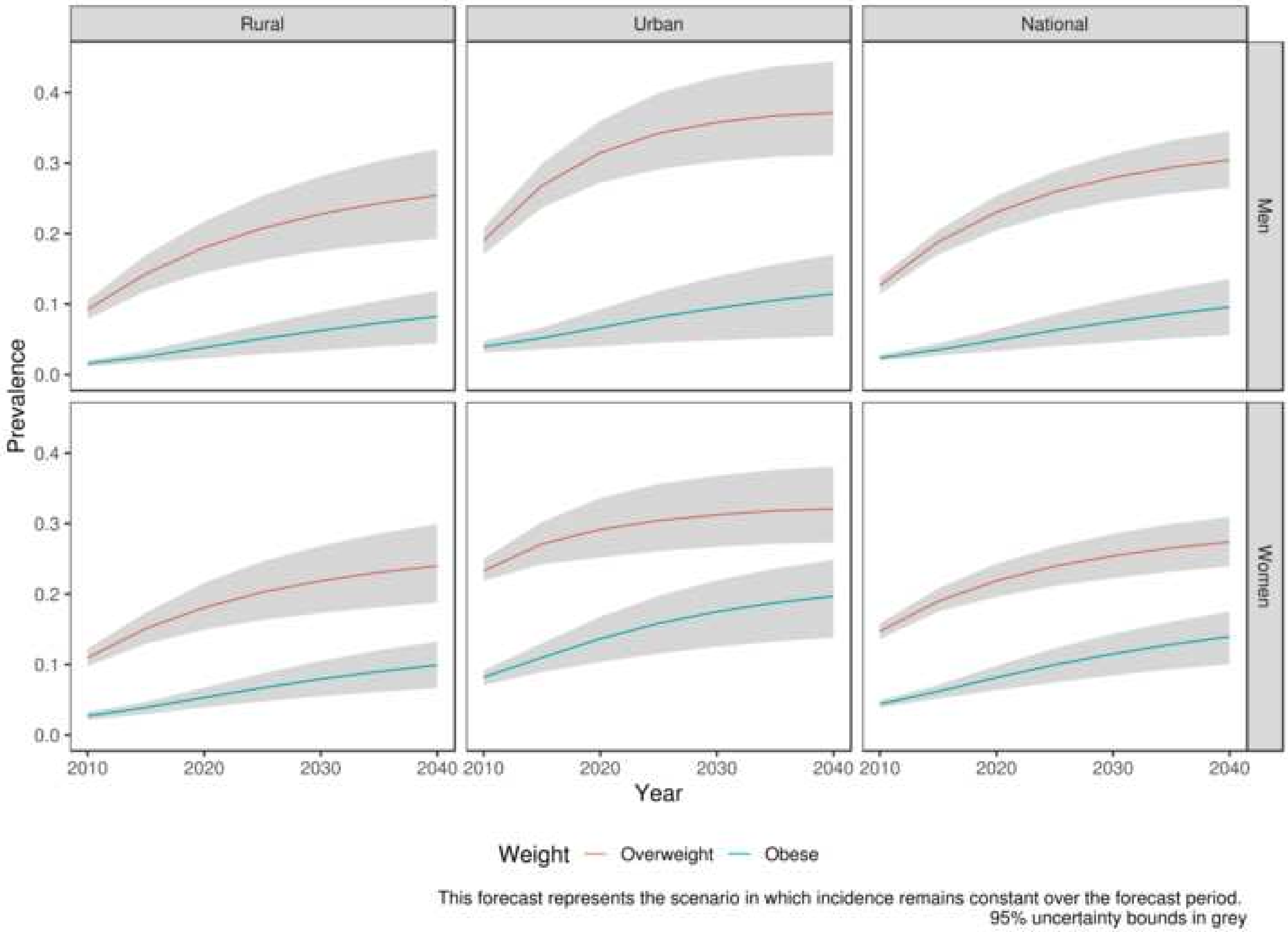

Figure 3

Click here to download Figure Figure 3.tiff

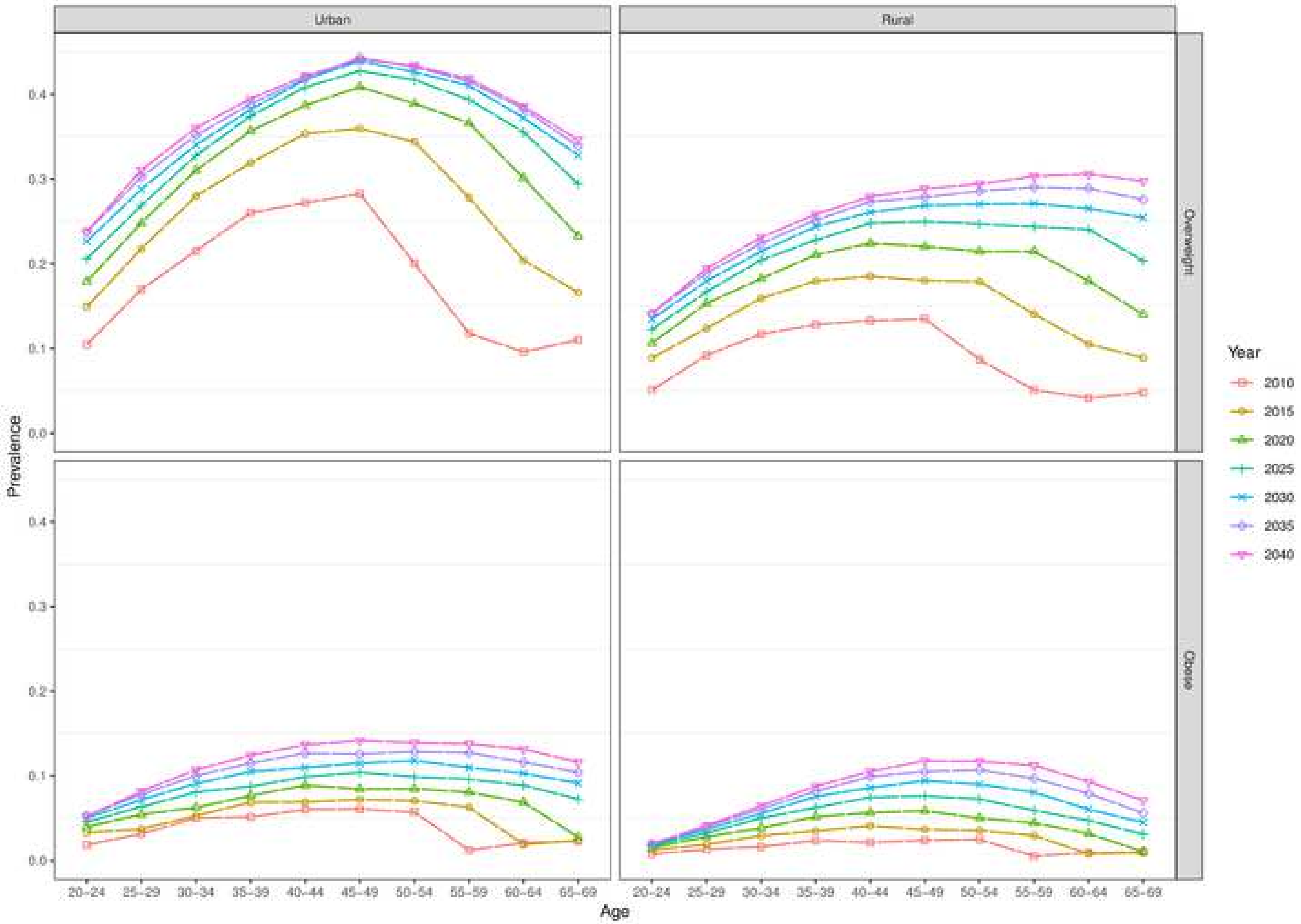

Figure 4

Click here to download Figure Figure 4.tiff

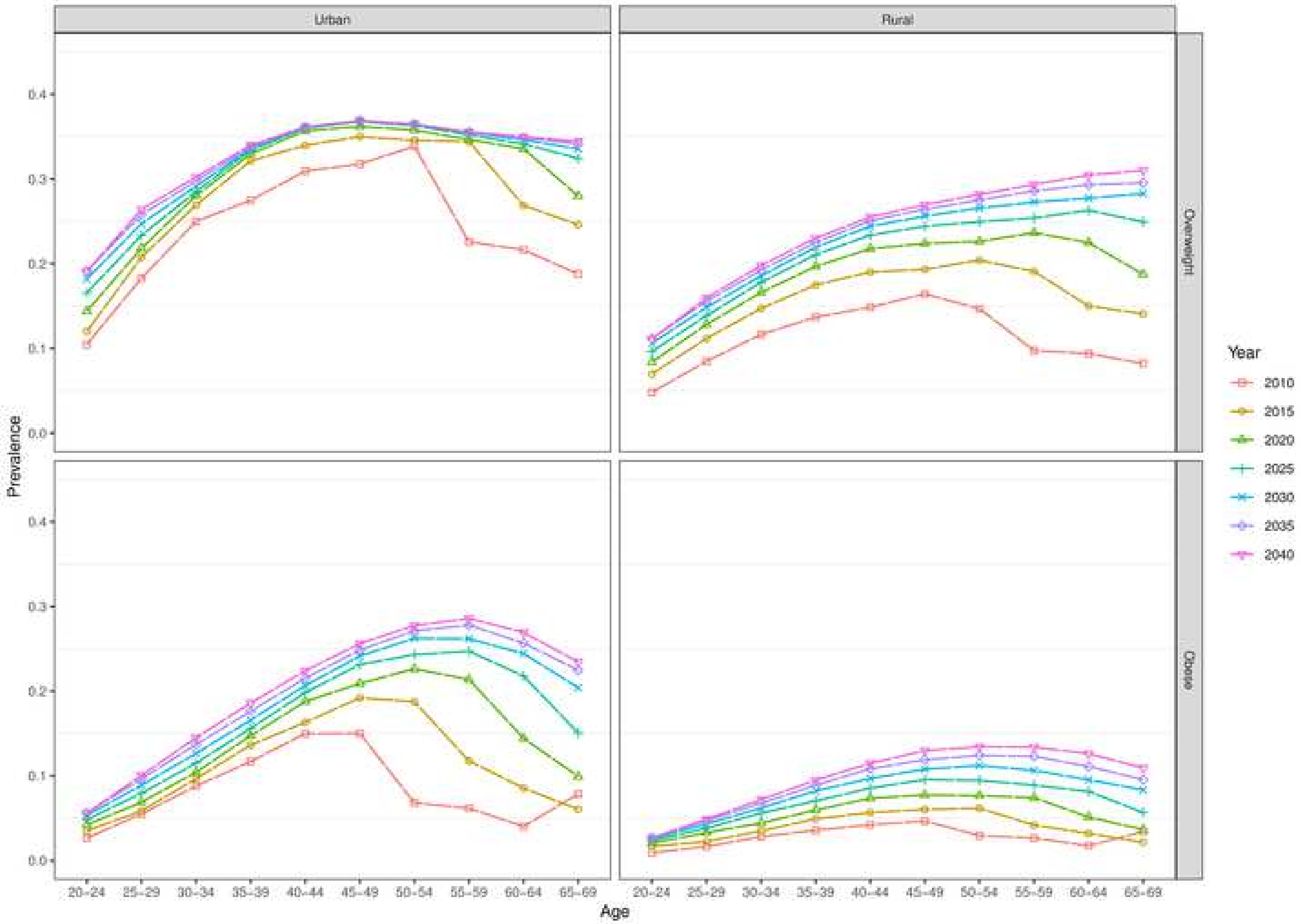

Figure 5

Click here to download Figure Figure 5.tiff

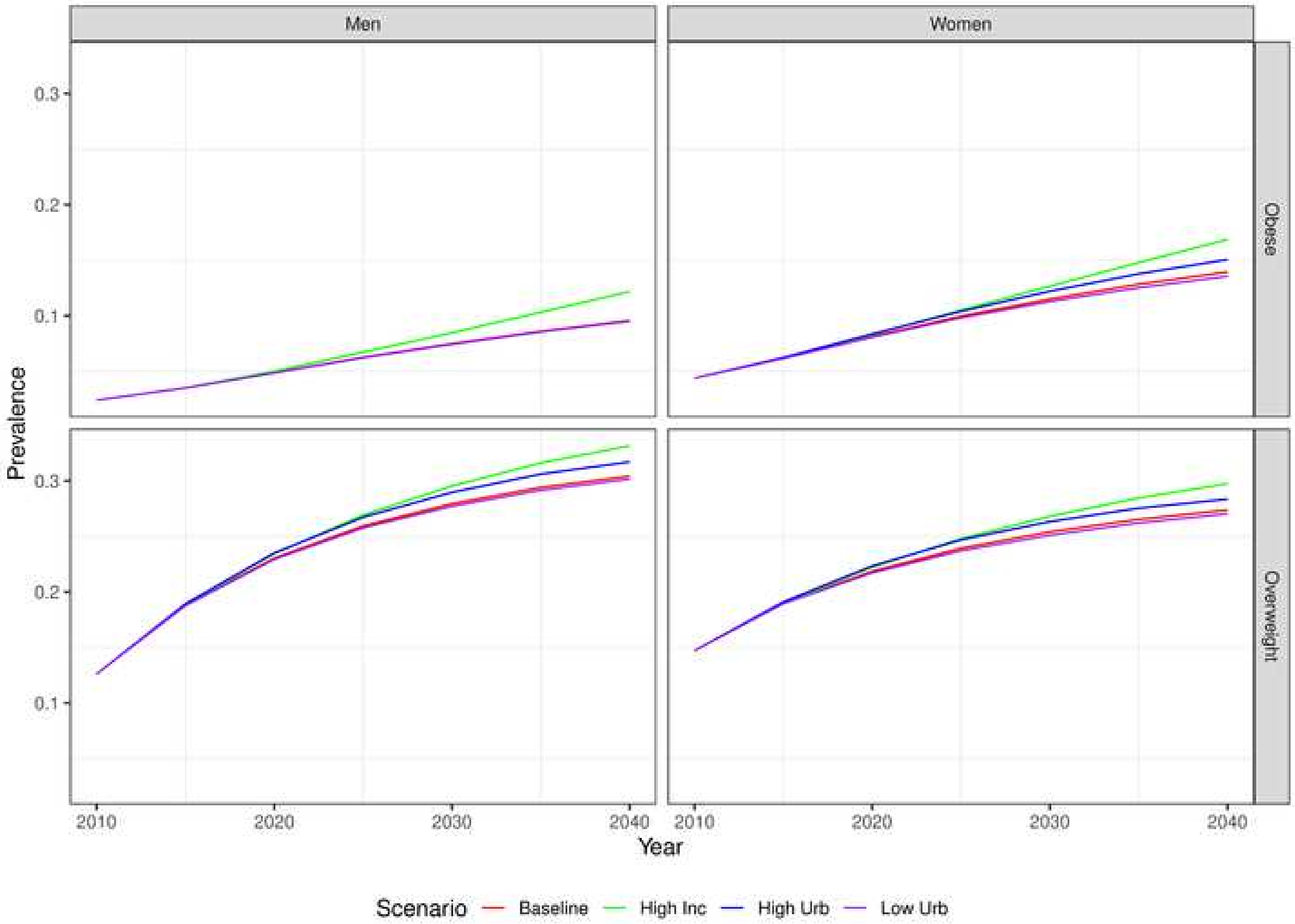

Appendix I-V

Click here to access/download
Supporting Information
Luhar et al PLoS Appendix.docx
