# Supplementary material for: Forecasting the prevalence of overweight and obesity in India to 2040
Source: PLoS One. 2020 Feb 24;15(2):e0229438. doi: 10.1371/journal.pone.0229438 (PMC7039458; doi:10.1371/journal.pone.0229438)
Supplement: Attachment — Submitted filename: PLoS One Response to Reviewers.docx [file pone.0229438.s004.docx]

Dear Editors,

We thank the reviewers for the comments given on the manuscript titled “**Forecasting the Prevalence of Overweight and Obesity in India to 2040”** We have edited the manuscript in Track Changes to address their concerns. Below we provide detailed responses to how we have addressed each of the comments. Additionally, the manuscript has been edited to match the journal’s preferred style.

We believe that the manuscript is now suitable for publication in PLoS One.

Best wishes,

Shammi Luhar

On behalf of all authors

***Reviewer 1***

1) The descriptive analyses related with prevalence of obesity for the years considered (From NFHS data and SAGE) for projection exercise can be included before presenting the results. Moreover, results can be presented for 5-year age groups as existing burden is higher for 40+ age groups.

We agree with the reviewer on this point and have reported the prevalence of overweight and obesity from the data sets in S2 Files. We chose to include this here rather than in the manuscript so as to not clutter the paper. In the section describing the input parameters, we have included the following on page 6 so that readers know they can refer to the Supporting Material for this information:

“The prevalence estimates from the data are included in the S2 File”.

2) A table on comparison of results from projection exercise with that of previous studies for India, their methodology and limitations can be included. The estimated burden can be presented for all the studies with the author’s calculation.

We agree with the reviewer. We have included a table in the S2 File that compares the results of the other forecasts of overweight and obesity in India, along with their limitations.

3) A look at the NFHS-4 data reveals that the prevalence of obesity is higher among the upper wealth quintiles. Therefore, projecting the burden separately by wealth quintiles might be a better idea for both rural and urban areas. The risk factor being adjusted for calculating obesity will vary across these groups. The results might change.

Wealth quintiles reported in survey data is a relative measure, and consequently, the definition of what it means to be in each quintile is likely to vary considerably over time. This, therefore, makes its inclusion in the model inappropriate in the author’s opinion.

The inclusion of a comparative wealth index may be a solution to the issue of comparability over time. However, the association of wealth with overweight and obesity is both complicated, extremely variable by various population subgroups, and has constantly changed over the past two decades. Additionally, the proportion of the population in each quintile of a comparative wealth index is likely to change considerably over time. We refer you to the article below that describes the trends in the association of socioeconomic position with overweight and obesity, and the extent of this variation in these trends sub-nationally^1^. Its inclusion, we believe, would add many extra layers of uncertainty that would detract from the straightforward and relatively elegant model we sought to build.

1. Luhar, S., Mallinson, P.A.C., Clarke, L. and Kinra, S., 2019. Do trends in the prevalence of overweight by socio-economic position differ between India’s most and least economically developed states?. *BMC Public Health*, *19*(1), p.783.

4) There is too much variation in BMI levels across States. The percentage of men and women who are overweight is surprisingly low in the most populated States such as Uttar Pradesh and Bihar. Given the dietary habits and pace of urbanization, it might not be desirable to project overall burden with assumptions that urbanization will grow at a stagnant rate.

We take this opportunity to clarify our use of the term urbanization in the context of this study. In this study, we used urbanization to refer to an individual’s propensity to migrate from a rural area to an urban area, and thus do not assume that urbanization will grow at a stagnant rate.

As urbanization is commonly defined as an increase in the proportion of the total population living in urban areas, assuming that the individual propensity to migrate to urban areas will remain constant over time implies that the growth in the urban population, whilst substantial, will increase at a variable rate. With this assumption, in states with a lower baseline prevalence of overweight/obesity and lower proportion urban, the rate at which the urban population will continue to grow will be faster than in states that have a higher proportion urban at baseline.

Moreover, use of low BMI thresholds for Asia can be one of the scenarios for the sensitivity analysis.

We agree with the reviewer’s comments that including sensitivity using the BMI cut-off values specific to South Asian populations are desirable. This sensitivity analysis is included in the S2 File, and is also referred to in the text on page 11:

“We also performed additional analysis using the South Asian BMI cut-offs values. Some advocate the use of these BMI cut-offs due to a stronger positive association between BMI and body fat observed in South Asians compared to White Caucasians, and consequently an elevated disease risk at lower BMI levels^2,3^. Under this assumption, a BMI between 23.00 kg/m^2^ and 27.49 kg/m^2^ was used to define individuals who are overweight, and a BMI greater than 27.50 kg/m^2^ was used to define obesity^4^.”

We have also included the following on page 17, regarding the limitation of using global-cut-offs:

“We sought to remedy this limitation by performing sensitivity analysis using South-Asian BMI cut-offs, and identified potential underestimation of our results (refer to S2 File). For instance, among urban men, we identified a potential underestimation of the 2040 obesity prevalence of nearly 20 percentage points, suggesting that using global cut-offs may underestimate the future overall public health challenge related to excess weight in India.”

***Reviewer 2***

- Regarding the paragraph on remission it is not clear to me what you mean by this. Please explain what is exactly meant with rates of remission.

We agree with the reviewer that this point could benefit from further clarification. In the text we have included the following on page 7:

“Remission refers to reverse transitions, whereby the simulated population is able to transition from a state of ‘Obese’ to ‘Overweight’, and ‘Overweight’ to ‘Not Overweight/Obese.”

- I do not understand the meaningfulness of the comparison of future prevalence rates in India to that of the US (page 19), as obesity rates in the US are already higher also at the baseline. Please explain why you are making this comparison.

The way in which I have phrased this is misleading due to my use of the word baseline (which refers to the baseline scenario and not the baseline year). I wanted to convey that even by 2040, India’s prevalence of obesity will not reach the prevalence levels that are currently observed in the United States or other industrialized countries, allowing potential for further increases in future prevalence in India. The sentence has been since altered to the following:

“Our study has found that the prevalence of obesity in 2040 is expected to be lower than levels that are currently observed in some of the world’s most industrialized economies, implying potential for considerable further increases beyond 2040.”

-Increases in overweight/obesity do not only result in higher prevalence rates of associated diseases but also in the need for medical care and facilities, which is in the short run more important, as it seems unrealistic with regard to past trends that the trends in overweight and obesity can be reversed in the short term. This could be better elaborated in the discussion.

We agree with the reviewer and have included the following in the discussion to elaborate on this point:

“The demand for medical services to tackle the increasing burden of overweight/obesity related diseases is also likely to increase substantially into the near future. Potential interventions include preventative measures such as screening for diabetes among high risk overweight/obese individuals to increase the proportion of people with diabetes that are diagnosed^5^. Further efforts may also wish to improve the provision of already established initiatives, particularly the National Programme for Prevention and Control of Cancer, Diabetes, Cardiovascular Disease and Stroke (NPCDCS), that in-part aims to reduce out of pocket expenditure on diabetes healthcare and promote behavioural and lifestyle improvements that reduce the risk of such diseases^6^.”

1.	Luhar, S., Alice, P., Mallinson, C., Clarke, L. & Kinra, S. Do trends in the prevalence of overweight by socio-economic position differ between India ’ s most and least economically developed states ? 1–12 (2019).

2.	Misra, A. Ethnic-Specific Criteria for Classification of Body Mass Index: A Perspective for Asian Indians and American Diabetes Association Position Statement. *Diabetes Technol. Ther.* **17**, 667–71 (2015).

3.	Stegenga, H., Haines, A., Jones, K. & Professor, J. W. Identification, assessment, and management of overweight and obesity: Summary of updated NICE guidance. *BMJ* **349**, g6608 (2014).

4.	Nishida, C. *et al.* Appropriate body-mass index for Asian populations and its implications for policy and intervention strategies. *Lancet* **363**, 157 (2004).

5.	Basu, S. *et al.* The Health System and Population Health Implications of Large-Scale Diabetes Screening in India: A Microsimulation Model of Alternative Approaches. *PLoS Med.* **12**, 1–21 (2015).

6.	Ministry of Health and Family Welfare, G. of I. National Program for Prevention and Control of Cancer, Diabetes, CVD and Stroke( NPCDCS). *Directorate General of Health Services* Available at: https://dghs.gov.in/content/1363_3_NationalProgrammePreventionControl.aspx. (Accessed: 14th August 2019)
