# Supplementary material for: Forecasting the prevalence of overweight and obesity in India to 2040
Source: PLoS One. 2020 Feb 24;15(2):e0229438. doi: 10.1371/journal.pone.0229438 (PMC7039458; doi:10.1371/journal.pone.0229438)
Supplement: Attachment — Submitted filename: Response_to_Reviewers_Shammi .docx [file pone.0229438.s005.docx]

Dear Editors,

We thank the reviewers for the further comments given on the manuscript titled “**Forecasting the Prevalence of Overweight and Obesity in India to 2040”.** We have since edited the manuscript in ‘Track Changes’ to address their concerns, and also attached a file with no ‘Track Changes’. Below, we provide detailed responses to how we have addressed each of the comments. Additionally, we have slightly amended the first supplementary file that details the methods used in the study. Specifically, we have made the methodology of the calculation of overweight and obesity incidence easier to follow and have made the tables more aesthetically pleasing.

We look forward to your response.

Best wishes,

Shammi Luhar

On behalf of all authors

Reviewer #1: I still do not agree with the assumptions that have been made with respect to urbanization and wealth.

We thank the reviewer for their comment and agree with the reviewer that forecasting the future prevalence of overweight and obesity by separate wealth quintiles would be ideal, however we opted against doing this for the following reasons:

Firstly, there are inherent inadequacies of a wealth index that is commonly used to capture socioeconomic position in cross sectional surveys in low- and middle-income countries. A wealth index is designed as a relative measure of wealth, rather than an absolute measure. Therefore, the index used to calculate quintiles is specific to the setting and the time period in which it is collected^1^. Consequently, it cannot be compared over time, and thus cannot be used in the forecasting of overweight and obesity over the specified forecast period.

Additionally, the changing nature of the association of overweight and obesity with socioeconomic position makes it very difficult to predict in the future which would be set externally to the model. Whilst it is true that survey data shows a higher prevalence of overweight and obesity in higher wealth quintiles, we are unable to firstly determine the proportion of the population that is expected to be in pre-specified wealth quintiles over the forecasting period (2010-2040), and secondly, it is not possible to ascertain what the exact association between overweight/obesity and socioeconomic position will be in the future. A number of systematic reviews^2–5^, in addition to recent studies in India^6–8^, have documented and provided evidence that in low and middle income countries, like India, the socioeconomic patterning of overweight and obesity changes considerably over time. The higher prevalence initially observed among high socioeconomic status groups will turn negative along with continued economic development. Due to the likely uncertain nature and considerable subnational heterogeneity in the future socioeconomic patterning of overweight and obesity throughout the forecasting period, we strongly believe that its inclusion in the model would be inappropriate and what results would come out of the model would be uninterpretable due to escalating uncertainty in the data and projections of SES distribution.

We have also included the following on page 18 under the limitations section:

“Finally, it would have been desirable to have accommodated the changing socioeconomic patterning of overweight and obesity in India in our forecasts. Studies have shown that rural and lower SEP Indians are at increasing risk of overweight and obesity, with significant variation in this patterning sub-nationally[43],[44]. However, due to the uncertainty in how these socioeconomic patterning trends will evolve throughout the forecasting period, the inclusion of socioeconomic status in our model has the potential to, at best, make the predictions marginally more accurate, and at worst, considerably more uncertain, and consequently, we opted for a relatively parsimonious model.”

References

1.	Rutstein SO, Johnson K. DHS Comparative reports No.6. The DHS Wealth Index. Calverton, Maryland, USA; 2004.

2.	Dinsa GD, Goryakin Y, Fumagalli E, Suhrcke M. Obesity and socioeconomic status in developing countries: A systematic review. Obes Rev. 2012;13(11):1067–79.

3.	McLaren L. Socioeconomic status and obesity. Epidemiol Rev. 2007;29(1):29–48.

4.	McLaren L. Socioeconomic status and obesity. Epidemiol Rev. 2007;29(1):29–48.

5.	Monteiro CA, Moura EC, Conde WL, Popkin BM. Public Health Reviews Socioeconomic status and obesity in adult populations of developing countries : a review. Public Health Rev. 2004;82(12):940–6.

6.	Luhar S, Mallinson PAC, Clarke L, Kinra S. Trends in the socioeconomic patterning of overweight/obesity in India: A repeated cross-sectional study using nationally representative data. BMJ Open. 2018;8(10):e023935.

7.	Luhar S, Alice P, Mallinson C, Clarke L, Kinra S. Do trends in the prevalence of overweight by socio-economic position differ between India ’ s most and least economically developed states ? 2019;1–12.

8.	Sengupta A, Angeli F, Syamala TS, Dagnelie PC, Schayck CP van. Overweight and obesity prevalence among Indian women by place of residence and socio-economic status: Contrasting patterns from “underweight states” and “overweight states” of India. Soc Sci Med. 2015;138:161–9.

In regards urbanisation, this study does not make any assumption that urbanisation (the proportion of the population living in urban areas) will continue at a stagnant rate. In the paper, we use the term ‘urbanisation’ to refer to any one individual’s propensity to migrate to an urban area, not the population level of urbanisation, which is commonly defined as an increase in the proportion of the population residing in urban areas.

The highly populated and low overweight/obesity states such as Uttar Pradesh and Bihar also have relatively lower proportion of the population in urban areas. In these states, with comparatively lower levels of overweight and obesity, the increase in the proportion of the population residing in urban areas will be relatively fast compared in these low overweight/obesity prevalence areas compared to high overweight/obesity prevalence areas, addressing the reviewer’s initial concerns.

Reviewer #1: Also, the process to arrive the burden is not clear.

We agree with the reviewer that the calculation of the burden was not clear. We have clarified this in the S2 File in a table footnote under the ‘Table of Comparison’ tab. Specifically, we included the following:

“For consistency across the different studies, we calculated the burden using the United Nations World Population Prospects (2017) population projections, and United Nations World Urbanization Prospects (2018), and applying the proportions identified in the studies”

Reviewer #2: The points raised in my first review have adequately been addressed. The authors have clarified what they mean with remission. The comparison to the US overweight and obesity rates makes sense now. However, the last sentence regarding future potential of obesity beyond 2040 is still a bit misleading. I would suggest not to call it "potential" but e.g. "vulnerability" or "susceptibility". The discussion section has also been elaborated.

We thank the reviewer for clarifying that we have adequayely addressed what was meant by remission. We agree with the reviewer’s additional concerns and have amended the sentence in the manuscript.

The original sentence:

“Our study has found that the prevalence of obesity in 2040 is expected to be lower than levels that are currently observed in some of the world’s most industrialized economies, implying the potential for considerable further increases beyond 2040.”

Has been changed to:

“We have found that the prevalence of obesity in 2040 is expected to be lower than levels that are currently observed in some of the world’s most industrialized economies, implying that India could be susceptible to considerable further increases in obesity prevalence beyond 2040.”
